# Insights Into the Evolutionary History of the Subfamily Orthotrichoideae (Orthotrichaceae, Bryophyta): New and Former Supra-Specific Taxa So Far Obscured by Prevailing Homoplasy

**DOI:** 10.3389/fpls.2021.629035

**Published:** 2021-03-26

**Authors:** Isabel Draper, Ricardo Garilleti, Juan Antonio Calleja, Maren Flagmeier, Vicente Mazimpaka, Beatriz Vigalondo, Francisco Lara

**Affiliations:** ^1^Centro de Investigación en Biodiversidad y Cambio Global, Madrid, Spain; ^2^Departamento de Biología (Botánica), Facultad de Ciencias, Universidad Autónoma de Madrid, Madrid, Spain; ^3^Departamento de Botánica y Geología, Facultad de Farmacia, Universidad de Valencia, Burjassot, Spain

**Keywords:** phylogeny, Zygodonteae, Orthotrichinae, Lewinskyinae, *Atlantichella*, *Australoria*, character evolution

## Abstract

Mosses of the subfamily Orthotrichoideae represent one of the main components of the cryptogam epiphytic communities in temperate areas. During the last two decades, this taxonomical group has undergone an extensive revision that has led to its rearrangement at the generic level. However, their phylogenetic relationships and inferences on the evolutionary patterns that have driven the present diversity have little advanced. In this study, we present a dated molecular phylogenetic reconstruction at the subfamily level, including 130 samples that represent the 12 genera currently recognized within the subfamily, and the analysis of four molecular markers: *ITS*2, *rps*4, *trn*G, and *trn*L-F. We also analyze 13 morphological characters of systematic value to infer their origin and diagnostic utility within the subfamily. The phylogenetic reconstruction yields three main clades within the subfamily, two of which correspond to the tribe Zygodonteae, and one to Orthotricheae. Within Zygodonteae, the genus *Zygodon* results to be a polyphyletic artificial assembly, and we propose to separate a new genus named *Australoria*. Conversely, our results do not support the separation of *Pentastichella* and *Pleurorthotrichum* at the genus level and we therefore propose to include *Pleurorthotrichum* in *Pentastichella*. Regarding Orthotricheae, our analyses clearly allow the distinction of two subtribes: Orthotrichinae and Lewinskyinae. Within the latter, *Ulota* results a polyphyletic entity, and therefore we propose the segregation of a separate new genus named *Atlantichella*. Dating analyses allow us to conclude that the split of the tribes within Orthotrichoideae dates from the Middle Jurassic, while the diversification of *Orthotrichum* and *Zygodon* probably started during the Late Cretaceous. However, most of the extant genera of this subfamily seem to be younger, and apparently its highest diversification burst took place during the Oligocene. Finally, the analysis of the morphological traits reveals that most of the characters previously used to separate genera and here tested are homoplastic, which has hindered the taxonomical and systematic proposals for decades. However, even if there are no exclusive characters, all of the genera can be defined by the combination of a few characters.

## Introduction

There is evidence that bryophytes have existed for more than 450 million years (Retallack, 2020). Their taxonomic diversity and phylogenetic relationships, as well as the evolution of relevant morphological characters, are still unclear, and there is a continuous renewal on their knowledge, especially thanks to the input of molecular tools (e.g., Coudert et al., 2017; Liu et al., 2019). Their diversification processes are nowadays becoming clearer, showing a remarkable variation with both patterns of prolonged stasis and/or rapid diversification rates in relation to climatic changes and interactions with other organisms such as vascular plants (e.g., Feldberg et al., 2014; Liu et al., 2014).

Despite the extensive information recently generated on the diversity and evolution of some groups of bryophytes, there are lineages still incompletely known. This is the case of the moss family Orthotrichaceae Arn., one of the most diverse among Bryophyta, with circa 900 species distributed in 25 genera (Goffinet and Vitt, 1998; Goffinet et al., 2004; Frey and Stech, 2009; Plášek et al., 2015; Lara et al., 2016). It comprises two subfamilies, Orthotrichoideae Broth. and Macromitrioideae Broth., which have very different morphological, biogeographic and ecological traits (Goffinet and Vitt, 1998; Goffinet et al., 1998; Frey and Stech, 2009; Lara et al., 2014). Macromitrioideae is almost exclusively intertropical, whereas Orthotrichoideae is characteristic of temperate and cold regions of both hemispheres, as well as of high altitudes in tropical mountains. In temperate areas, Orthotrichoideae is one of the main components of the epiphytic communities, both in dry (Draper et al., 2006; Lara et al., 2009) and in oceanic or hyper-oceanic conditions (Garilleti et al., 2015; Lara et al., 2016).

During the last two decades, the subfamily Orthotrichoideae has undergone an extensive taxonomical revision that has included relevant generic rearrangements (Table 1). At the end of the last century, Goffinet and Vitt (1998) published a revised classification based on the combination of their analyses of morphological characters, with the revision and compilation of the previous knowledge on Orthotrichaceae. They included 11 genera in Orthotrichoideae, separated into two tribes: Zygodonteae Engler, and Orthotricheae Engler. A new genus segregated from *Orthotrichum* Hedw. was soon added to this list: *Sehnemobryum* Lewinsky-Haapasaari & Hedenäs (Lewinsky-Haapasaari and Hedenäs, 1998[1999]). Conversely, *Orthomitrium* Lewinsky-Haapasaari & Crosby was later considered a synonym of *Orthotrichum* (Allen, 2002).

**TABLE 1:** Evolution of the classification of Orthotrichoideae during the last two decades.

Goffinet and Vitt, 1998	Goffinet et al., 2004	Plášek et al., 2015	**Current proposal**
**A) Tribes and genera recognized by the mentioned authors and dates.**			
**Tribe ORTHOTRICHEAE**	**ORTHOTRICHEAE**	-	**ORTHOTRICHEAE**
***Orthotrichum*** Hedw.	***Orthotrichum*** Hedw.	***Orthotrichum*** Hedw.	***Orthotrichum*** Hedw.
***Muelleriella*** Dusén			
***Orthomitrium*** Lewinsky & Crosby			
	***Nyholmiella*** Holmen & E.Warncke	***Nyholmiella*** Holmen & E.Warncke	***Nyholmiella*** Holmen & E.Warncke
***Stoneobryum*** D.H.Norris & H.Rob.	***Stoneobryum*** D.H.Norris & H.Rob.	-	***Stoneobryum*** D.H.Norris & H.Rob.
	***Sehnemobryum*** Lewinsky & Hedenäs	-	***Sehnemobryum*** Lewinsky & Hedenäs
		*Dorcadion* Lindb. *nom. illeg.*	***Lewinskya*** F.Lara, Garilleti & Goffinet
		***Pulvigera*** Plášek, Sawicki & Ochyra	***Pulvigera*** Plášek, Sawicki & Ochyra
***Ulota*** D.Mohr	***Ulota*** D.Mohr	***Ulota*** D.Mohr	***Ulota*** D.Mohr
		***Plenogemma*** Plášek, Sawicki & Ochyra	***Plenogemma*** Plášek, Sawicki & Ochyra
			***Atlantichella*** F.Lara, Garilleti & Draper

**Tribe ZYGODONTEAE**	**ZYGODONTEAE**	-	**ZYGODONTEAE**
***Zygodon*** Hook. & Taylor	***Zygodon*** Hook. & Taylor	-	***Zygodon*** Hook. & Taylor
*Stenomitrium* (Mitt.) Broth. *nom. illeg.*	***Pentastichella*** Müll.Hal.		***Pentastichella*** Müll.Hal.
***Pleurorthotrichum*** Broth.	***Pleurorthotrichum*** Broth.		
			***Australoria*** F.Lara, Garilleti & Draper
***Bryomaltaea*** Goffinet			
***Leptodontiopsis*** Broth.			
***Codonoblepharon*** Schwägr.	***Codonoblepharon*** Schwägr.	-	***Codonoblepharon*** Schwägr.

**B) Transferences of genera and erections by other authors in the period**			
***Sehnemobryum*** Lewinsky & Hedenäs (Lewinsky-Haapasaari and Hedenäs, 1998[1999])			
*Orthomitrium* Lewinsky & Crosby - Transferred to ***Orthotrichum*** (Allen, 2002)			
*Muelleriella* Dusén - Transferred to ***Orthotrichum*** (Goffinet et al., 2004)			
*Bryomaltaea* Goffinet - Transferred to ***Leratia* Broth. & Paris** (Macromitrioideae) (Goffinet et al., 2004)			
*Leptodontiopsis* Broth. - Transferred to ***Zygodon*** (Goffinet et al., 2004)			
***Lewinskya*** F.Lara, Garilleti & Goffinet (Lara et al., 2016)			
*Pleurorthotrichum* Broth. - Transferred to ***Pentastichella*** (current proposal)			

This concept of the subfamily changed a few years later thanks to the first study based on molecular data from several loci (Goffinet et al., 2004). It provided genetic support to the systematic treatment of the subfamily and indicated that several of the genera traditionally conceived were paraphyletic. To accommodate the taxonomy, Goffinet et al. (2004) proposed the synonymization of the genera *Bryomaltaea* Goffinet, *Leptodontiopsis* Broth., and *Muelleriella* Dusén (Table 1B), and recognized the genus *Nyholmiella* Holmen & E. Warncke as a separate entity. Their molecular data revealed other incongruences with the traditional classification based on morphological characters, which evidenced the need of further studies. The most intriguing results were that *Orthotrichum* and *Zygodon* Hook. & Tayl. were not resolved in monophyletic clades, as well as the lack of information to infer the relationships among *Pentastichella* Müll.Hal., *Pleurorthotrichum* Broth., and *Zygodon*.

One decade later, Plášek et al. (2015) proposed a new subdivision of *Orthotrichum* and *Ulota* D. Mohr into three and two genera respectively (Table 1), which was later sustained by additional genomic data (Sawicki et al., 2017; Mizia et al., 2019). Hence, the currently accepted classification of Orthotrichoideae comprises a total of 12 genera (Table 1).

Despite the number of recently published contributions on the generic classification of Orthotrichoideae and its taxonomical diversity at the species level (among others: Caparrós et al., 2011; Medina et al., 2012; Wang and Jia, 2012; Caparrós et al., 2016; Fedosov and Ignatova, 2018; Vigalondo et al., 2019a; Lara et al., 2020), few advances have been made regarding its phylogenetic relationships and evolutionary patterns. Thus, the proposal of Goffinet et al. (2004) remains the most complete phylogeny so far published, including 22 taxa that represent 9 of the 12 genera now recognized in Orthotrichoideae. Other papers have included the reconstruction of partial phylogenies of the subfamily, yet they included a small fraction of species or focused on the resolution of species complexes within certain genera (e.g., Sawicki et al., 2009; Sawicki et al., 2017; and Lara et al., 2020).

The time framework of the evolutionary history of the subfamily Orthotrichoideae remains currently ignored. It is well known that dating molecular phylogenies in Bryophyta is challenging due to the scarce fossil record. In the case of Orthotrichoideae, the closest fossil record is a member of Macromitrioideae (Heinrichs et al., 2013), which is a poor dating calibration point in a phylogeny of the subfamily Orthotrichoideae. However, the lack of fossils can be overcome by applying substitution rates taken from related plant groups or by other combined approaches (see Villarreal and Renner, 2014 for a review). In the case of Bryophyta, Laenen et al. (2014) published the up to date most complete dated phylogeny, reporting ages for several Orthotrichoideae genera, yet understood in a broad sense (*Ulota* sensu lato, *Zygodon* s. l., *Orthotrichum* s. l., and *Pentastichella*), that does not contribute to understand the diversification of the subfamily into the current taxonomical framework at genus level (Table 1, second and third columns). Additionally, there have been only two attempts to recover diversification times on Orthotrichoideae, but limited to different species complexes (Patiño et al., 2013; Vigalondo et al., 2019b).

Together with the diversification time-frame, the knowledge on the evolutionary history of one group is enhanced by the analysis of the evolution of significant morphological characters. In this sense, numerous studies have analyzed the evolution of key traits within mosses, such as genome size (Bainard et al., 2020), gametophyte branching (Coudert et al., 2017), sporangium shape (Rose et al., 2016), sexual system in correlation with life-history traits (Crawford et al., 2009), ancestral characters related to habitat preferences (Huttunen et al., 2018), or perichaetium position in pleurocarpous mosses (Bell and Newton, 2007). In general, these studies yield conclusions above the family level that are sometimes applicable at a lower taxonomical scale, but usually lack a sufficient representation of the morphological variability at the genus level. Within Orthotrichoideae, Lewinsky-Haapasaari and Hedenäs (1998[1999]) performed a very complete cladistic analysis including 83 morphological characters and 129 species. Their results led them to draw conclusions on the possible evolution of *Orthotrichum* s. l. and they hypothesized about the most probable traits of the ancestor of the group. Unfortunately, the cladistic method strictly based on morphology seems to lack consistent phylogenetic significance. Among the few molecular phylogenies of Orthotrichaceae, only Goffinet et al. (2004) drew some inferences on character evolution, such as the reduction of the chromosome number in the subfamily, although they did not include an analysis of morphological states in their work.

The studies based on integrative taxonomy published during the last decades (e.g., Medina et al., 2012, 2013; Caparrós et al., 2016; Vigalondo et al., 2016, 2019a; Lara et al., 2020) suggest that morphological characters are valid for an efficient distinction of the species in Orthotrichoideae even in complexes with great morphological similarities. However, the same morphological traits frequently fail to establish phylogenetic relationships because similar or cryptic species are often not closely related in sister clades. In this study, we intend to verify whether the pattern observed at the species level is repeated at the supra-specific level. In this sense, our hypothesis is that supra-specific taxa in Orthotrichoideae are not characterized by exclusive morphological features (autapomorphies).

In order to verify this hypothesis, this study moves forward on the following aims: 1) to provide an updated phylogeny of the subfamily Orthotrichoideae, including all the genera currently accepted and an accurate taxa representation within the tribe Orthotricheae; 2) to analyze the diversification process of the subfamily within a molecular time calibrated framework; and 3) to infer the systematic utility of the main key morphological characters for the differentiation of the genera currently accepted.

## Materials and Methods

### Taxa Sampling

A total of 130 samples have been included in the molecular analyses, which represent the 12 genera currently accepted within Orthotrichoideae (Table 1, second and third columns). Special emphasis was given to the sampling of the tribe Orthotricheae, with circa 50% of the accepted species (Frey and Stech, 2009; Garilleti et al., 2015; Lara et al., 2016): *Orthotrichum* (42 samples that belong to 39 species, out of the about 100 accepted), *Stoneobryum* D. H. Norris & H. Rob. (one sample of one of the two accepted species), *Ulota* (29 samples of 24 species, out of about 70 accepted), *Nyholmiella* (two of two currently accepted), *Sehnemobryum* (one sample of the single recognized species), *Lewinskya* F.Lara, Garilleti & Goffinet (33 samples of 32 species, out of the about 70 recognized), *Pulvigera* Plášek, Sawicki & Ochyra (four samples of the four recognized species), *Plenogemma* Plášek, Sawicki & Ochyra (four samples of the single recognized species), *Codonoblepharon* Schwägr. (three samples of two species, out of the seven recognized), *Pleurorthotrichum* (one sample out of the single recognized species), *Pentastichella* (one sample of the one recognized species), and *Zygodon* (seven samples of seven species, out of the about 90 recognized). In addition, one sample of *Leratia obtusifolia* (Hook.) Goffinet and one of *Macrocoma lycopodioides* (Schwägr.) Vitt have been included as outgroup according to the results by Goffinet et al. (2004). Details on the samples included are shown on Supplementary Appendix 1.

A wide selection of additional specimens from MAUAM was included in the morphological study. The purpose of this was the codification of the morphological characters for the estimation of the ancestral states (see section “Analysis of Key Morphological Traits”) as well as the reinforcement of the conclusions that could be derived from the phylogenetic analyses.

### DNA Isolation, Amplification, and Sequencing

DNA was extracted for 26 of the 130 samples included in the molecular analyses. The rest of the samples were previously included in other phylogenetic studies and their DNA extraction process has been published elsewhere (Supplementary Appendix 1). DNA was extracted from the tip of single gametophyte shoots using the DNeasy Plant Mini Kit for DNA isolation (Qiagen). The rest of the gametophyte, together with the capsule of the sporophyte, was preserved in a microscope slide fixed with glycerogelatin to allow for a re-identification, if needed.

Four molecular loci were amplified by PCR, three from the plastome (*rps*4, *trn*G and *trn*L-F) and one from the nuclear genome (the nuclear internal transcribed spacer II, *ITS*2). The primer pairs used for each of these loci were respectively rpsA (Nadot et al., 1994)/trnaS (Souza-Chies et al., 1997); trnGF-Leu (Stech et al., 2011)/trnGr (Pacak and Szweykowska-Kulinska, 2000); trnLc-104/trnFF-425 (Vigalondo et al., 2016); and ITS2-F/ITS2-R (Ziolkowski and Sadowski, 2002). The PCRs were performed using Ready-To-Go PCR Beads (Amersham Pharmacia Biotech Inc) in a final reaction volume of 25 μl, according to the manufacturer’s instructions, with 2–4 μl of template DNA. The protocol for the *rps*4 consisted on one cycle of 5 min at 94°C, 30 cycles of 30 sec at 95°C, 1 min at 52°C, and 30 sec at 68°C, and one final cycle of 7 min at 68°C. For the *trn*G the protocol used was one cycle of 5 min at 94°C, 40 cycles of 30 sec at 95°C, 40 sec at 52°C, and 1 min and 30 sec at 72°C, and one final cycle of 8 min at 72°C. For the *trn*L-F the protocol consisted on one cycle of 5 min at 95°C, 38 cycles of 30 sec at 94°C, 1 min at 47°C, 30 sec at 72°C, and 30 sec at 94°C, and one final cycle of 10 min at 72°C. For the *ITS*2 the protocol was one cycle of 1 min at 94°C, 30 cycles of 1 min at 94°C, 1 min at 59°C, and 1 min and 30 sec at 72°C, and one final cycle of 5 min at 72°C. PCR products were purified using Exo/SAP (Thermo Fisher Scientific, Spain) in a mixture of 1 μl of Exo1 enzyme and 4 μl of FastAP enzyme per 25 μl of PCR product, following the protocol indicated in the manufacturer’s instructions. Cleaned PCR products were sequenced by Macrogen^1^.

### Phylogenetic Analyses

The obtained forward and reverse reads were visually checked and aligned to create the consensus sequences using Geneious v. 9.1.8^2^. Initially, one independent matrix was created for each locus using PhyDE 0.9971 (Müller et al., 2006). The 3’ and 5’ ends were trimmed in order to eliminate uncertain base pairs that could undermine the resolution of the results. Trimming included the following base pairs: in *ITS*2, the primers plus 90 bp at 5’/50 bp at 3,’ in *rps*4, the primers plus 49/58 bp, in *trn*G, the primers plus 25/75 bp, and in *trn*L-F, the primers at the two ends plus 18 bp at the 3’ end. The resulting matrices were automatically aligned using Mafft multiple sequence alignment tool at EMBL-EBI (Madeira et al., 2019) under the E-INS-i iterative method. Insertions and deletions (indels) in non-coding regions are sometimes difficult to assess and can lead to ambiguous alignments. To determine the effect of their inclusion, all analyses were run three times: with the indels considered as missing information, the indels coded as informative characters, and on reduced matrices where the indel blocks were deleted. The indel coding strategy was the simple method of Simmons and Ochoterena (2000), as implemented in SeqState (Müller, 2004). The reduced matrices were obtained with Gblocks v. 0.91b (Castresana, 2000; Talavera and Castresana, 2007).

The congruence among the independent matrices was tested with the ILD test in TNT v. 1.1 (Goloboff et al., 2003). Incongruences were not detected and we created a combined matrix with the information of the four loci. The best evolutionary model and partition scheme to fit the molecular data was selected with PartitionFinder v.2.1.1 (Lanfear et al., 2016), resulting in three subsets (*ITS*2, *rps*4 and *trn*L-F + *trn*G) with the model GTR + I + G for *ITS*2 and *trn*L-F + *trn*G, and the model GTR + G for *rps*4. The phylogenetic relationships were analyzed under Bayesian Inference (BI) and Maximum Likelihood (ML). Bayesian Inference was performed using MrBayes v. 3.2.7 (Huelsenbeck and Ronquist, 2001; Ronquist and Huelsenbeck, 2003) at CIPRES (Miller et al., 2010). We ran four chains for 4 000 000 generations, sampling trees and parameters every 1000th. The convergence and stability of the analysis was analyzed in Tracer v. 1.7.1 (Rambaut et al., 2018). Posterior probabilities (PP) were estimated for the 50% majority rule consensus tree after a burn-in of 25% of the generated trees. Maximum Likelihood analyses were conducted in MEGA X (Kumar et al., 2018), with an initial tree obtained by the Neighbor-Joining method to a matrix of pairwise distances estimated using the Maximum Composite Likelihood (MCL) approach. The resulting trees were visualized and edited in FigTree v.1.4.4 (Rambaut, 2018).

### Divergence Times Estimation

Bayesian inference was also used to estimate divergence times, as implemented in BEAST v. 1.10.4 (Suchard et al., 2018) at CIPRES (Miller et al., 2010). Twenty-seven different models were performed (Supplementary Appendix 2). For six of these models, the combined data matrix was divided in two partitions, one corresponding to the nuclear DNA and the second one corresponding to the plastid DNA, to be able to apply different substitution rates for the two genomes. These were fixed at 1.35 × 10^–3^ substitutions per site per Myr for the *ITS* as estimated by Bakker et al. (1995), and 5.0 × 10^–4^ substitutions per site per Myr for the plastid set as estimated by Palmer (1991) and Sanderson (2002). The rest of the analyses were run with a constant substitution rate along the combined matrix, that according to previous literature was fixed at 5.0 × 10^–4^ substitutions per site per Myr, as estimated by Palmer (1991) and Sanderson (2002). Further details on the options chosen are shown in Supplementary Appendix 2. Each model was run for 80 000 000 generations to achieve an adequate effective sample size. To assure this, the results of the BEAST runs were analyzed in Tracer v. 1.7.1 (Rambaut et al., 2018). Marginal likelihood estimations were computed for each model using both path sampling (PS) and stepping-stone sampling (SS). To calculate these, each model was run four times, and the marginal likelihood estimation for each model was calculated as the mean of the values obtained on the four runs. The model with the highest mean marginal likelihood was selected as the best to fit our data. The results obtained from the four runs of the selected model were combined with LogCombiner v. 1.10.4 (Drummond and Rambaut, 2007), with a burn-in of 25% of the generated trees, and resampling every 1000th. The consensus tree of the trees obtained from the combination of the results of the four runs of the best model was created with TreeAnnotator v. 1.10.4 (Drummond and Rambaut, 2007) and visualized and edited in FigTree v.1.4.4 (Rambaut, 2018).

As previously indicated, a fossil record is lacking for Orthotrichoideae. Thus, divergence times estimated by Laenen et al. (2014) were used as calibration points for the analyses. These authors included seven species of Orthotrichoideae in their broad sampling of Bryophyta, which allowed us to include two of their time estimations as reference points: the age of *Pentastichella* and the divergence time of *Ulota* and *Orthotrichum*. According to the results of the phylogenetic analyses, monophyletic taxon sets were created for *Lewinskya*, *Orthotrichum*, *Pentastichella*, *Pulvigera*, *Ulota*, and *Zygodon*, as well as for two big clades within Orthotrichoideae (see below). According to Laenen et al. (2014), the estimated age for *Pentastichella* was set to 9.37 Ma [1 – 26] and the separation of the two big clades within Orthotrichoideae that respectively include *Orthotrichum* and *Ulota* was set to 133.62 Ma [91 – 183].

### Analysis of Key Morphological Traits

Thirteen discrete morphological characters of both the gametophyte and the sporophyte were scored for all of the samples included in the molecular phylogeny (Supplementary Appendix 3). This selection comprises the traits that *a priori* show a relevant variation within Orthotrichoideae, and especially among Orthotricheae, but does not intend to study the character evolution at the family level. The 13 selected characters entail a systematic meaning in the studied tribe. Nevertheless, their phylogenetic significance is variable, and can be summarized as follows:

(1) Three characters are widely considered of systematic value in the group: a) growth form (creeping or erect); (b) sexual condition (dioicous or monoicous); and (c) stomata architecture (immersed or superficial).
(2) Six traits vary within the genera, although in some cases they characterize one genus or a species group. Because of this, they have been previously considered of systematic value by some authors, at least at the sub-generic level: (a) leaf position when dry (crisped or erect to slightly sinuose); (b) dimorphic longitudinal bands of basal cells (present or absent); (c) brood bodies (present or absent); (d) endostome connective membrane (present or absent); (e) capsule striation (smooth or ribbed); and (f) spores multicellular (present or absent).
(3) Finally, the four remaining traits are generally considered to be unique to one genus or even a single species (i.e., autapomorphic), so they have not been previously analyzed in an evolutionary context: (a) submarginal band of elongate cells (present or absent); (b) basal marginal cells differentiated (*Ulota* type) (present or absent); (c) basal margins with geminate teeth (*Pulvigera* type) (present or absent); (d) flagelliform branches (present or absent).

To infer the possible origin of these selected characters, ancestral states were estimated using Mesquite v. 3.61 (Maddison and Maddison, 2019). The input tree used to plot the characters was the one obtained from the analysis of the combined matrix (*ITS*2-chloroplast-gaps coded as informative) by BI in MrBayes, with the selected parameters described above. In addition, the characters were also plotted on an ultrametric tree to assess whether the tree topology influences the ancestral states reconstruction, according to the results of Cusimano and Renner (2014). The results obtained both using the phylogram and the ultrametric tree were identical, therefore the ultrametric results are not shown. In absence of previous information regarding the probability of change from one state to another, the type of analysis performed to infer the ancestral states was ‘trace characters history’ by the most parsimonious reconstruction method. Characters were treated as unordered categorical variables.

## Results

### Molecular Phylogenetic Reconstruction

**FIGURE 1:**
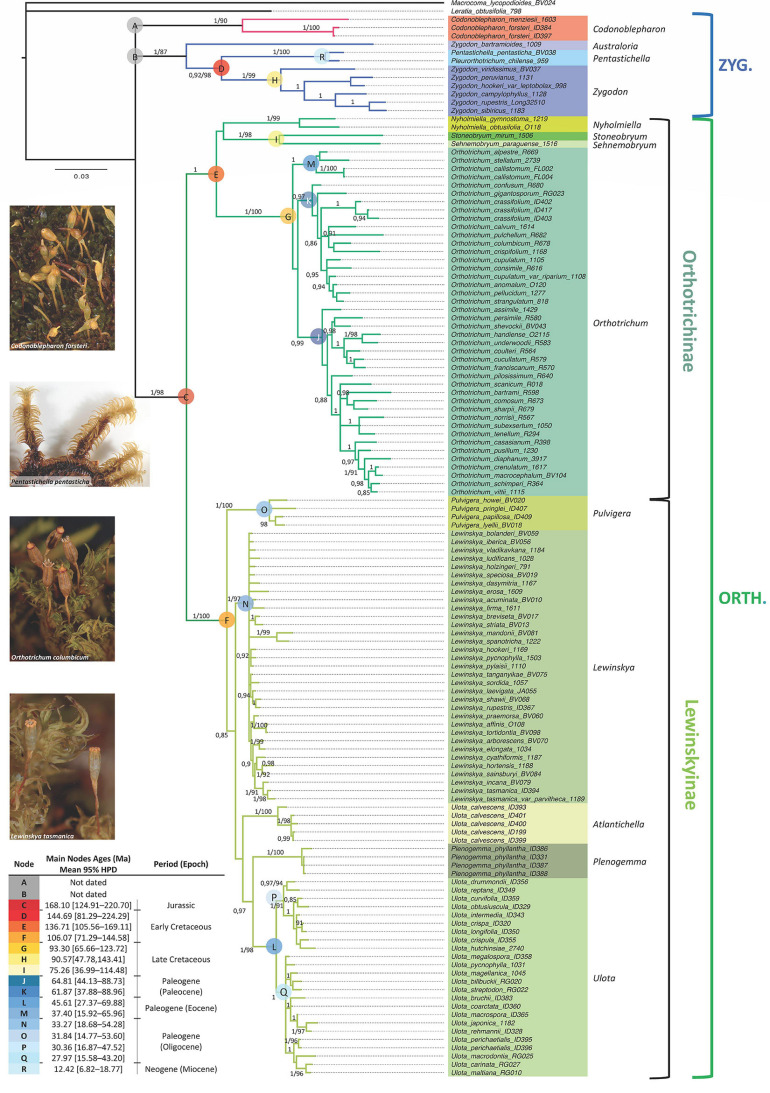
Majority-rule consensus tree obtained in the Bayesian Inference analysis of the combined data matrix (*ITS*2, *rps*4, *trn*L-F, *trn*G and indels coded). Bayesian Posterior probabilities (≥ 0.85) and Maximum Likelihood bootstrap values (≥ 85%) are shown above the branches. Sequences information is given in Supplementary Appendix 1. Molecular dating corresponds to the results obtained from the analysis under a relaxed uncorrelated log-normal clock with Yule speciation and an absolute nucleotide substitution rate of 5 × 10^–4^, with stdev. range of 1.5 × 10^–4^.

The concatenated molecular data matrix of *ITS*2, *rps*4 and *trn*L-F + *trn*G had a total length of 2128 bp, of which 551 sites were parsimony informative. The codification of the indels added 168 parsimony informative characters. Information about the number of variable and informative sites of each data partition is shown in Table 2. The different analyses performed led to congruent results for the ingroup clades in all cases (phylogenetic reconstruction on each single locus matrix, indels treated as missing data, codified as additional characters or deleted, and Bayesian and Maximum Likelihood methods). The phylogenetic reconstruction shown in Figure 1 corresponds to the analysis by Bayesian Inference of the concatenated data matrix, with gaps coded and included as informative characters, which resulted in the best resolved and had the highest clade support of all the analyses performed. The subfamily Orthotrichoideae is divided into three groups (clades A, B and C). Clade C corresponds to the tribe Orthotricheae, which results monophyletic and well supported (1/98). However, the representatives of Zygodonteae as traditionally conceived are divided into two well-supported clades, one including the samples of *Codonoblepharon* (A; 1/90) and the other one including *Zygodon*, *Pentastichella* and *Pleurorthotrichum* (B; 1/87). The sister relationships among these two clades and Orthotricheae are not resolved in our phylogeny.

**TABLE 2:** Number of variable and potentially informative sites for the partitions defined in the molecular data matrix.

	***ITS*2**	***rps*4**	***trn*G-*trn*L-F**
Variable sites (ingroup)	284	154	371
Variable sites (total)	299	156	408
Informative sites (ingroup)	209	94	227
Informative sites (total)	218	99	234
Indel sites	141	3	149
Indel informative sites (ingroup)	73	0	87
Indel informative sites (total)	81	0	87
Positions in data matrix	1–506	507–1034	1035–2128

Our results indicate that *Zygodon* as currently understood is paraphyletic because *Zygodon bartramioides* Dusén ex Malta results to be a well separated taxonomical entity in all the analyses. This taxon is basal to a clade (D) that comprises the rest of the samples of *Zygodon*, together with *Pentastichella* and *Pleurorthotrichum*. These latter two genera are closely related on the well-supported clade R (1/100). The rest of the species of *Zygodon* also constitute a well-supported monophyletic clade (clade H, hereafter *Zygodon* s. str.; 1/99).

Within clade C, there is a clear division into two subclades: one (E) including *Orthotrichum*, *Nyholmiella*, *Stoneobryum* and *Sehnemobryum*, and the other (F) including *Pulvigera*, *Lewinskya*, *Ulota*, and *Plenogemma*. All these genera except *Ulota* constitute monophyletic well-supported clades. Regarding *Ulota*, samples of *U. calvescens* Carrington are gathered in a monophyletic clade (1/100) that is clearly separated from the clade that gathers the rest of *Ulota* species (clade L, hereafter *Ulota* s. str.; 1/98).

The relationships among the different genera within clades E and F are not fully resolved in our phylogeny. *Stoneobryum* and *Sehnemobryum* are related as sister genera in clade I (1/98), but the sister relationship of this clade with *Nyholmiella* or *Orthotrichum* is poorly supported and differs between the ML and BI results. Regarding the group of genera gathered in clade F, *Pulvigera* is located in a basal position in all the analyses, sister of a moderately supported clade (0.85/–) that contains the remaining genera. Within this big clade, BI results provide high support (0.97/–) for the sister relationship of *Ulota* s. str. (clade L) with *Plenogemma*, but the sister relationship of these two with either the *Ulota calvescens* or *Lewinskya* clades is poorly supported.

Regarding the genetic variation in the main genera, our results show low genetic variation within *Lewinskya*, but clearly differentiated subclades are defined within *Orthotrichum* (three subclades: J, K, and M) and *Ulota* s. str. (two subclades: P and Q).

### Divergence Times Estimation

The best model for dating the obtained phylogeny is the relaxed uncorrelated log-normal clock with a Yule speciation tree-prior (Supplementary Appendix 2). The original chronogram obtained from the analysis is shown in Supplementary Appendix 4, and estimated ages for the main clades are included in Figure 1. The nucleotide substitution rate applied (5 × 10^–4^, with standard deviation range of 1.5 × 10^–4^) establishes a mean age of 168.10 Ma for clade C (Figure 1, 95% highest posterior density interval (HPD): [124.91 – 220.70 Ma]), which corresponds to the tribe Orthotricheae, dating its diversification in the Middle Jurassic. The mean age estimated for clade D (that corresponds to Zygodonteae excluding *Codonoblepharon* and *Zygodon bartramioides*) is 144.69 Ma (95% HDP: [81.29 – 224.29 Ma]), corresponding to the Jurassic-Cretaceous boundary. Our results do not allow a safe estimation of the age of the clade including *Codonoblepharon* because this genus is only represented by two species in the phylogeny and it only has been possible to amplify all the molecular markers for one of them. The same problem applies to estimate the age of *Z. bartramioides*.

Among the genera within Zygodonteae, *Zygodon* s. str. (clade H) seems to be from the Late Cretaceous, with a mean age of 90.57 Ma (95% HDP: [47.78 – 143.41 Ma]), whereas the closely related clade R that gathers *Pentastichella* and *Pleurorthotrichum* dates from Neogene (Miocene), with a mean age of 12.42 Ma (95% HDP: [6.82 – 18.77 Ma]).

Regarding Orthotricheae, *Orthotrichum* (clade G) also results to be a relatively old genus from the Late Cretaceous, with a mean age of 93.30 Ma (95% HDP: [65.66 – 123.72 Ma]). *Ulota* s. str. (clade L; mean age of 45.61 Ma, 95% HDP: [27.37 – 69.88 Ma]), *Lewinskya* (clade N; mean age of 33.27 Ma, 95% HDP: [18.68 – 54.28 Ma]) and *Pulvigera* (clade O; mean age of 31.84 Ma, 95% HDP: [14.77 – 53.6 Ma]) would have been diversified along the Paleogene. *Ulota* is probably from the Eocene whereas *Lewinskya* and *Pulvigera* could be from the Oligocene. According to the MP analysis, *Plenogemma* shares a common ancestor with *Ulota* s. str., but this relationship is not supported by the IB analysis and the separation of these two genera could not be dated. Similarly, the sister relationship of *Nyholmiella* with either clade I (*Stoneobryum* and *Sehnemobryum*) or clade G (*Orthotrichum*) remains unclear, and without a robust age estimation. Likewise, the relationships of the clade that comprises *Ulota calvescens* specimens is yet poorly resolved.

### Analysis of Morphological Traits

**FIGURE 2:**
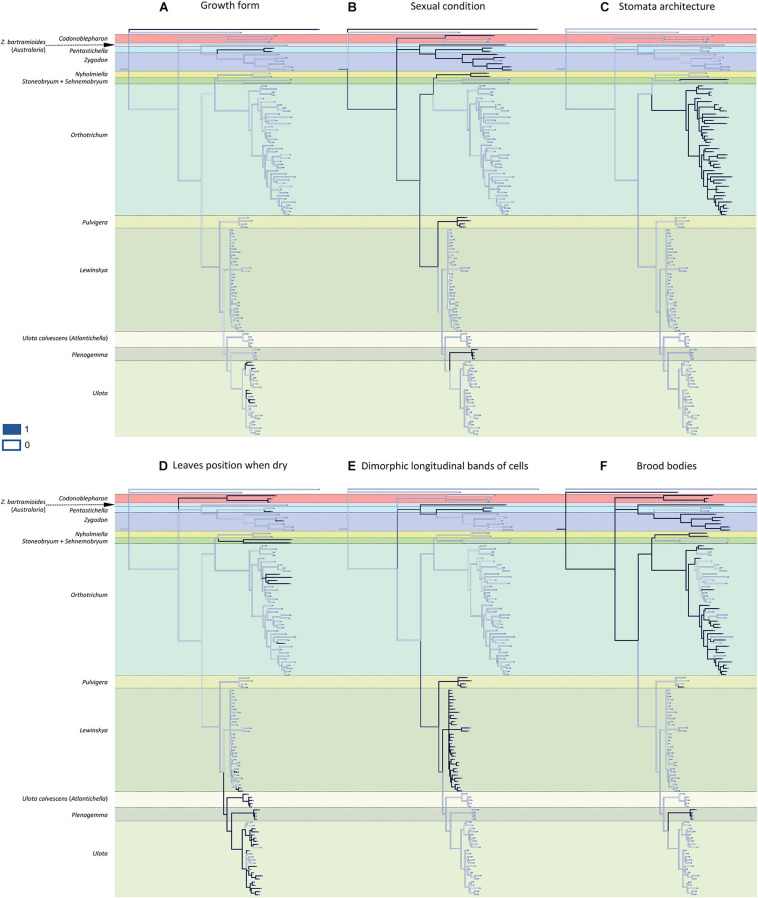
Reconstruction of ancestral states for the selected characters performed by Mesquite on the BI tree shown in Figure 1: **(A)** growth form (1: creeping growth, 0: erect growth); **(B)** sexual condition (1: dioicous, 0: monoicous); **(C)** stomata architecture (1: immersed, 0: superficial); **(D)** leaves position when dry (1: crisped, 0: erect to slightly sinuose); **(E)** dimorphic longitudinal bands of basal cells (1: present, 0: absent); **(F)** brood-bodies (1: present, 0: absent).

**FIGURE 3:**
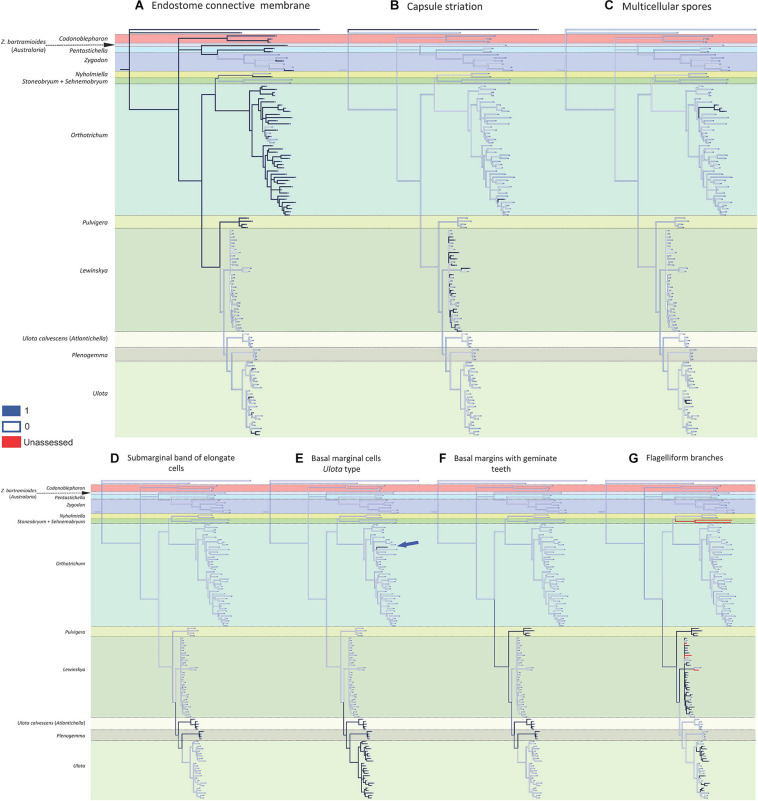
Reconstruction of ancestral states for the selected characters performed by Mesquite on the BI tree shown in Figure 1: **(A)** endostome connective membrane (1: present, 0: absent); **(B)** capsule striation (1: smooth, 0: ribbed); **(C)** multicellular spores (1: present, 0: absent); **(D)** submarginal band of elongated cells (1: present, 0: absent); **(E)** basal marginal cells of leaves differentiated (*Ulota* type) (1: present, 0: absent); **(F)** basal margins with geminate teeth (*Pulvigera* type) (1: present, 0: absent); **(G)** flagelliform branches (1: present, 0: absent).

Most of the 13 morphological characters analyzed are homoplastic (Figures 2, 3), although seven of them can be used as diagnostic: 1) dioicous sexual condition is present in one of the two representatives of *Codonoblepharon* included in the analyses (clade A in Figure 1), in all *Zygodon* species except *Z. hookeri* var. *leptobolax* (Müll.Hal.) Calabrese, in *Pentastichella* and *Pleurorthotrichum* (all in clade B), and in all the species of *Nyholmiella*, *Pulvigera*, and *Plenogemma* (clade C); 2) stomata are immersed only in *Orthotrichum* and *Stoneobryum* (clade E); 3) dimorphic longitudinal bands of basal cells have been verified in *Zygodon bartramioides* (clade B), *Pentastichella* and *Pleurorthotrichum* (clade R), *Pulvigera* and *Lewinskya* (clade F); 4) multicellular spores are only present in one member of *Orthotrichum* (*O. crassifolium* Hook.f. & Wilson) (clade K), and two of *Ulota* (*U. billbuckii* Garilleti, Mazimpaka & F.Lara and *U. streptodon* Garilleti, Mazimpaka & F.Lara) (clade Q), among all the included representatives of these genera; 5) submarginal band of elongated cells is restricted to one *Ulota* species (*U. calvescens*), and the one corresponding to *Plenogemma* (both in clade F); 6) basal marginal leaf-cells differentiated as in *Ulota* are characteristic of the clade that encompasses almost all the species of *Ulota* (clade L), although the character is also present in *Ulota calvescens* and *Plenogemma* (clade F), and, surprisingly, in one species of *Orthotrichum* (*O. calvum* Hook.f. & Wilson) (clade K); and 7) basal marginal cells with geminate teeth (*Pulvigera* type) are only present in *Ulota calvescens*, *Plenogemma* and *Pulvigera* (clade F).

The reconstruction of the ancestral states indicates that the ancestor of the Orthotrichoideae probably had neither creeping growth nor flagelliform branches. Its leaves likely were erect when dry, without submarginal bands of elongated cells or basal marginal cells differentiated, without geminate teeth in the basal marginal cells, and without dimorphic longitudinal bands of basal cells. Regarding the characters related to reproduction, the ancestor presumably developed brood-bodies. Our data do not provide enough evidence to conclude the sexual condition of the ancestor. Its sporophytes probably had smooth capsules, with superficial stomata and connective membrane in the endostome, and most likely developed unicellular spores.

## Discussion

### Phylogeny of Orthotrichoideae

Orthotrichoideae has traditionally been considered a taxonomically complex subfamily. The difficulties to differentiate many of the species of *Orthotrichum* s. l. are well known (Moxley, 1937; Lewinsky, 1993) and the same can be said for the other two major traditionally recognized genera *Ulota* and *Zygodon* (Calabrese, 2006; Caparrós et al., 2016). Moreover, the delimitation of supra-specific taxonomical categories in this group is also intricate, as can be deduced from the recent rearrangements proposed at the generic level (Table 1, Goffinet et al., 2004; Plášek et al., 2015). The up-to-date accepted generic classification of Orthotrichoideae is based on molecular phylogenies with a low representation of the taxonomical diversity within the group. The molecular phylogeny of the present study (Figure 1) includes a wide representation of all the genera currently accepted and its results have systematic implications. Firstly, the subfamily appears divided into three main clades (A, B, and C), which contrasts with the two previously recognized tribes (Goffinet et al., 1998). Clade C clearly corresponds to Orthotricheae, whereas Zygodonteae appears split into clades A and B. Clade A, currently only including *Codonoblepharon*, points toward the existence of a third independent lineage among Orthotrichoideae. However, the lack of support and the retrieved polytomy advise postponing the possible implications of these results until confirmed in further studies on a wider representation of species and biogeographical ranges.

Within clade B, the genus *Zygodon* is a polyphyletic artificial ensemble. Both the molecular phylogeny and its morphological features underpin that *Zygodon bartramioides* belongs to a separate taxonomical entity that can be recognized as a separate genus. To accommodate these results, we here propose a new genus named *Australoria* F.Lara, Garilleti & Draper (see section “The Genus *Australoria*” for its description). In addition, Zygodonteae currently includes the genera *Pentastichella* and *Pleurorthotrichum*. These two genera are closely related (clade R in Figure 1, and Figures 2, 3) and their segregation at the genus level remains unsupported. Moreover, morphological examination of their representatives shows that, despite their different appearance, both share a large number of significant characters that support their consideration as members of the same genus. Therefore, we propose to include *Pleurorthotrichum* in *Pentastichella*, which is the oldest name of the two. An amended description of this genus, including the variability derived from the inclusion of *Pleurorthotrichum*, is provided in section “The Genus *Pentastichella*.”

Regarding Orthotricheae, the phylogenetic results evidence two well defined clades that deserve the consideration of subtribes: Orthotrichinae F.Lara, Garilleti & Draper and Lewinskyinae F.Lara, Garilleti & Draper. Orthotrichinae includes four well established genera: *Orthotrichum*, *Sehnemobryum*, *Stoneobryum*, and *Nyholmiella*. As for Lewinskyinae, it comprises four genera: *Lewinskya*, *Pulvigera*, *Plenogemma*, and *Ulota*. However, our results prove the last of these genera to be polyphyletic. Hence, we propose the segregation of *Ulota calvescens* in a separate new genus named *Atlantichella* F.Lara, Garilleti & Draper, which is described in section “The Genus *Atlantichella*,” with its differential characters highlighted.

At the infrageneric level, our results do not support the traditional subgenera segregations within *Orthotrichum* (Vitt, 1973; Lewinsky, 1993). The molecular phylogeny shows three well-supported clades (named J, K, and M in Figure 1), but these clades do not correspond to the concept of any of the last proposed subgenera, and no morphological characters seem to support their segregation and description at this level. Similarly, within *Ulota* s. str. there are two well supported clades (named P and Q in Figure 1) that could correspond to subgenera, although a further study including additional samples would be needed to establish the morphological characters supporting this classification.

### The Genus *Australoria*

The genus *Australoria* is proposed to segregate *Zygodon bartramioides* from *Zygodon* s. str. on the basis of its phylogenetic position and its distinctive morphological features (Figures 1, 2). Both the studies by Malta (1926) and Calabrese (2006) evidenced that *Z. bartramioides* shows key morphological characteristics that separate it from *Zygodon* s. str., some of which are shared with *Pentastichella.* Our morphological study on recently collected specimens corroborates this perception and led to consider that the similar *Zygodon chilensis* Calabrese & F.Lara (Calabrese et al., 2006), which is not included in the present phylogenetic reconstruction, also belongs to this genus. *Australoria* is thus an endemic of the North Andean-Patagonia region (see distribution maps in Calabrese, 2006), and is characterized by the following traits:

Plants medium sized to robust, in compact turfs, with stems erect. Leaves spirally arranged to more or less distinctly in five rows, with base not sheathing, < 2 mm long; basal leaf cells rectangular, smooth, dimorphic, arranged in longitudinal bands alternating rows of hyaline, thin-walled cells, with rows of yellowish cells with thick and porose walls; median and upper leaf-cells papillose. Propagula usually abundant, cylindrical, sometimes branched at its ends. Dioicous. Perichaetial leaves differentiated. Seta > 2 mm, sinistrorse in the distal part. Capsule exserted, ovoid to ellipsoidal or cylindrical, symmetric, entirely ribbed. Exothecial bands differentiated, suboral ring of several rows of small exothecial cells differentiated. Stomata superficial at the capsule neck. Peristome double, with exostome of 8 pairs of robust teeth, partially splitting, recurved and appressed to the capsule when dry; endostome of 8 linear segments, sometimes with some less developed intercalary segments (8 + n), and a low basal membrane, continuous and striate. Spores foveolate (with numerous depressions or cavities), small, 10–12 μm in diameter, trilete mark distinct. Calyptra cucullate, naked. Male specimens of similar size to the female ones.

Among the diagnostic characters, *Australoria* shows three differential traits that can be highlighted for being absent or exceptional in the closely related *Zygodon* s. str.: a) the cylindrical sometimes branched propagules; b) the foveolate spores; and c) the presence of differentiated longitudinal bands of basal cells, a character shared with *Pentastichella* (Figure 2). This latter trait is also present in *Leptodontiopsis* (Goffinet and Vitt, 1998), a genus of erect, robust, dioicous plants, devoid of propagules, whose phylogenetic relationships need further studies since it is currently considered to be a synonym of *Zygodon* (Goffinet et al., 2004).

The two species recognized in *Australoria* can be differentiated by the following key characters:

– Leaves lanceolate-rhomboidal; propagules hyaline, with only transverse septa, not branched; seta 2–5 mm diameter – *A. bartramioides*
– Leaves lanceolate; propagules mostly colored, often with distal longitudinal septa or branched; seta ca. 10 mm diameter – *A. chilensis*

### The Genus *Pentastichella*

*Pleurorthotrichum* was accurately revised by Lewinsky-Haapasaari (1994), who concluded the inclusion of a single species, *P. chilense*, in this Chilean genus. A thorough and updated description of the Patagonian *Pentastichella pentasticha* (as *Zygodon pentastichus*, Calabrese, 2006) is also available. The morphological data in these two studies show that both taxa share multiple characteristics. However, they had not been previously related as members of the same genus. Our phylogenetic results (Figure 1) and the subsequent morphological study carried out support this taxonomic consideration and thus, *Pentastichella* is here recognized as including two species: *Pentastichella pentasticha* and *Pentastichella chilensis* (Broth.) F.Lara, Garilleti & Draper (≡ *Pleurorthotrichum chilense*). The morphological traits that define the genus are the following ones:

Plants robust, in loose tufts or mats, with stems both plagiotropic and erect, pentagonal star-shaped in section. Leaves arranged in five rows (pentastichous), with long sheathing base and a patent distal part, > 2 mm long; basal leaf cells strongly dimorphic, differentiated in longitudinal bands, with long and conspicuous colored bands of porose, thick-walled cells; median and upper leaf-cells papillose. Propagules absent. Dioicous. Vaginula strongly hairy. Perichaetial leaves well to strongly differentiated. Seta > 3 mm, sinistrorse in the distal part. Capsule emergent to exserted, oblong-cylindric, slightly asymmetric, entirely ribbed. Exothecial bands differentiated, suboral ring of several rows of small exothecial cells differentiated. Stomata superficial at the capsule neck. Peristome double, with 16 exostome partially paired teeth, reflexed when dry, and 16 endostome segments, arising from a more or less tall connecting membrane. Spores papillose, medium-sized to large, > 17 μm, with indistinct trilete mark. Calyptra cucullate, naked or hairy. Male specimens of similar size to the female ones, generally more compact, with somewhat shorter leaves. Perigonia terminal, both on the main axes, and on lateral branches, large, with abundant paraphyses and antheridia. Perigonial leaves with a broad base and a relatively short blade, yellowish to orange, the outer ones little differentiated from the vegetative leaves.

The two included species have a very distinct aspect, mainly due to the very different shape of the leaves, and differ from each other by additional key characters:

– Leaves up to 7 mm in length, long acuminate, loosely crisped, contorted to circinate when dry; perichaetial leaves very long and conspicuous; setae 3–4 mm long; calyptrae strongly hairy; endostome of thin segments, not blocking completely the mouth when dry; spores 30–45 μm in diameter – *P. chilensis*
– Leaves up to 3 mm in length, shortly acuminate, erect to patent, sometimes somewhat flexuose when dry; perichaetial leaves scarcely outstanding; setae 10–15 mm long; calyptrae naked; endostome of broad segments, occluding the mouth when dry; spores 17–28 μm in diameter – *P. pentasticha*

*Pentastichella pentasticha* shows a very peculiar peristome, being that the endostome is strongly developed, with a tall continuous connecting membrane, and broad segments that fully block the mouth when dry. However, similar structures can be found in *Matteria* Goffinet (Macromitrioideae), as well as in some species of *Ulota* and *Lewinskya*, and they have been related to adaptive strategies for the release of spores in particular epiphytic habitats (see Garilleti et al., 2012). On the other hand, *P. chilensis* shows a distinctive appearance on the basis of its peculiar leaves, very long and tortuous when dry, that are highly different to those of other Zygodonteae species. Its cucullate and hairy calyptrae also constitute an exceptional feature, although this character can be found in a few species of *Zygodon* s. str., even if cucullate naked calyptras absolutely predominate in the genus. Therefore, this morphological character should not be considered of systematic value.

### The Genus *Atlantichella*

The genus *Atlantichella* is proposed to accommodate *Ulota calvescens* on the basis of its unusual combination of morphological traits and phylogenetic position. The morphological characterization of the proposed genus is:

Plants medium sized, with stems erect, in cushions. Leaves spirally arranged, strongly crisped when dry, lanceolate, with base hardly differentiated, not or scarcely concave, often plicate on both sides of the nerve, with erect-incurved margins, leaf lamina unistratose, irregularly bistratose and mainly plane at margins; basal cells long rectangular to linear, with thick, somewhat sinuose to nodulose walls; basal-marginal cells differentiated, hyaline, quadrate to rectangular, with thickened transverse walls, forming a narrow marginal band along the base and proximal end of the lamina; margins at upper base with papillose teeth arising at the junctions between two cells; submarginal rows of elongated cells differentiated from base through lower third of the lamina; median and upper leaf-cells rounded to elliptical, with low papillae. Propagula absent. Autoicous. Perichaetial leaves slightly differentiated, larger, with a broader, somewhat sheathing base. Seta 3–6 mm long. Capsule exserted, ellipsoidal to cylindrical, symmetric, entirely ribbed, with a long neck, strongly contracted below mouth when dry. Exothecial bands broad, differentiated from mouth to urn base. Stomata superficial, at urn base and neck. Peristome double; remnants of a low protostome frequently present; exostome of 8 pairs of teeth, partially splitting after recurving; endostome of 8 linear segments, without connective membrane. Operculum with undulated base. Spores unicellular, isomorphic, papillose, 15–25 μm in diameter. Calyptra mitrate, covered with scarce to abundant stout hairs.

The morphological similarities of *Atlantichella* with *Plenogemma* and *Ulota* s.s. are numerous, sharing the same general aspect. Its morphological differentiation is based on the presence of a set of characters, of which none is completely exclusive. However, only *Atlantichella* has these two clear characters always combined: the autoicous sexual condition and the presence of leaves with submarginal bands of well-developed elongated cells.

### Diversification Time Framework in Orthotrichoideae

The diversification times that have been obtained in this study are significantly different from two of those previously published for the genera of Orthotrichaceae. The divergence times shown in Figure 1 and Supplementary Appendix 4 have been established by the combined use of an absolute nucleotide substitution rate for plastid and nuclear sequences (as estimated by Palmer, 1991, and Sanderson, 2002), plus secondary dating from Laenen et al. (2014) for *Pentastichella* (9.37 Ma, with 95% HPD [1 – 26 Ma]) and the segregation of Orthotrichinae and Lewinskyinae (133.62 Ma, with 95% HPD [91 – 183 Ma]). These authors included a sampling selection that allowed them to estimate the ages of *Zygodon bartramioides* (119.11 [90 – 167]) and *Orthotrichum* (104.16 [76 – 151]), in addition to the two estimations used as calibration points in our phylogeny. Our results are congruent with the ones that they obtained for *Orthotrichum*, since our estimation is slightly younger (93.30 [65.66–123.72]) and overlaps with the interval that they provided. However, our results for *Z. bartramioides* suggest an older separation of this species than the one provided by Laenen et al. (2014). As explained above, we failed to obtain sequences from all the molecular loci for the studied samples of *Z. bartramioides*, and therefore we could not estimate the age of this species. However, it appears to be a sister taxon of clade D in the phylogenetic reconstruction (Figure 1), which is dated in the Early Cretaceous (144.69 [81.29–224.29]).

Apart from Laenen et al. (2014), only two other studies have reported diversification times for Orthotrichoideae. Patiño et al. (2013) established a mean age of 33.64 Ma [22.71 – 46.53] for the clade containing the species of *Orthotrichum* s. str., in contrast to the 93.30 Ma [65.66 – 123.72] reported here. These authors used an average absolute substitution rates of chloroplast DNA, but they did not include explicit calibration points, which could explain the difference between their estimation and our results. On the other hand, Vigalondo et al. (2019b) dated the clade containing the species of *Orthotrichum* s. str. on 61.13 Ma [39.45 – 91.13], which overlaps the interval provided in our study, although it suggests a relatively younger diversification. Their method for the time estimation is similar to the one here applied, and we interpret that the different ages reported are probably a consequence of the different taxon sampling used in the two reconstructions. While Vigalondo et al. (2019b) studied the diversification date of one *Orthotrichum* species, our study focuses on a wider phylogenetic frame that includes a complete representation of all the genera of Orthotrichoideae, which probably has allowed for a better estimation of the age of the *Orthotrichum* clade.

The differentiation of the tribes Orthotricheae and Zygodonteae dates from the Middle Jurassic, when important biogeographic events such as the breakup of Gondwanaland took place. At that time, the landscape was probably dominated by conifers (Willis and McElwain, 2002). The diversification within *Orthotrichum* and *Zygodon*, as well as of the Lewinskyinae lineage, probably started during the Cretaceous, a period with a relatively warm climate, when flowering plants diversified and became widespread, extending into polar latitudes (Axelrod and Raven, 1978; Willis and McElwain, 2002; Magallón et al., 2015). This fact is crucial for Orthotrichaceae, since many species are epiphytes and therefore their potential habitats would have been remarkably increased, as it has been also inferred for epiphytic leafy liverworts (Feldberg et al., 2014). However, most of the extant genera of the subfamily Orthotrichoideae seem to be younger, and apparently, the highest diversification burst of the subfamily took place during the late Eocene and the Oligocene, which is considered to be a period of transition between more tropical to cooler conditions (Zachos et al., 2001; Willis and McElwain, 2002). The genera *Ulota*, *Lewinskya*, and *Pulvigera* probably originated in that period, which is characterized by the regression of humid and warm tropical forests and parallel increase of forested and open habitats with temperate climates (Zachos et al., 2001; Willis and McElwain, 2002), suitable for a relevant proportion of the species of these genera (Draper et al., 2006; Lara et al., 2009). The youngest genus (*Pentastichella*) would have originated during the Miocene, which is also considered a period of cool and dry climate. Extant species of this genus grow in two different situations of the pacific coast of southern South America: *P. chilensis* is restricted to very humid, mostly coastal locations under Mediterranean climate of the north-central Chile, whereas *P. pentasticha* extends through the northern Andean Patagonian region, reaching south central Chile. Their ancestor also could have thrived in humid and temperate areas of the extreme south of South America. As the climate became cooler and drier it would have taken refuge in both the warmer areas with a clearly hyperoceanic climate of northern Patagonia and in exceptionally humid refuges of the Mediterranean region. Subsequently, a process of speciation under allopatric conditions would have taken place.

Our results agree with Laenen et al. (2014), who concluded that certain lineages of bryophytes increased their diversification rates during the Cenozoic, and hypothesized that bryophytes are still actively diversifying. Similar results and diversification times were also obtained by Bell et al. (2015) and Pereira et al. (2019) for the moss families Polytrichaceae Schwägr. and Calymperaceae Kindb. respectively, and by Lee et al. (2020) for the liverwort genus *Lejeunea* Lib., all of them supporting the hypothesis of Laenen et al. (2014). Interestingly, Amo de Paz et al. (2011) also reported similar diversification rates for the parmelioid genera, the largest clade of macrolichens, which are also commonly found in epiphytic habitats. They dated the origin of Parmeliaceae Zenker in the late Cretaceous, although they stated that most of the parmelioid lineages originated during the Eocene cooling and the Oligocene glaciation, and indicated that the radiation of the current genera occurred during the Miocene. Therefore, two of the main components of the epiphytic communities in temperate climates (Parmeliaceae and Orthotrichoideae) share a similar diversification pattern, linked to climatic changes and to angiosperms radiation and expansion.

### Characterization of Key Morphological Traits in Orthotrichoideae

For a long time, Orthotrichoideae has been considered a complex subfamily that included three large genera (*Orthotrichum*, *Ulota*, and *Zygodon*), which showed a great inner variability in key morphological traits. The recent rearrangements at the generic level (Plášek et al., 2015) have partly clarified this treatment. As an example, *Orthotrichum* s. l. included species with both immersed and superficial stomata, but its segregation into *Orthotrichum* s. str. (cryptoporous) and *Lewinskya* (phaneroporous) implied the recognition of this morphological trait as diagnostic at the genus level (Lara et al., 2016). However, a general overview of the systematic utility of the main morphological characters used for Orthotrichoideae is lacking.

The present study reveals a generalized homoplasy in the analyzed traits since none of them is exclusive and characteristic of a single genus. Among the studied characters, three different patterns can be inferred. A) Seven traits are clearly homoplastic, having appeared several times and in separate lineages along the diversification of Orthotrichoideae: creeping growth, immersed stomata, leaves crisped when dry, dimorphic longitudinal cell bands, unribbed capsules, multicellular spores, and differentiated basal marginal cells like those of *Ulota* (Figures 2, 3). B) Three characters are probably synapomorphies and originated in a single ancestral occurrence that was followed by a subsequent loss of the trait in one or several lineages: presence of a submarginal band of elongated cells, basal margins with geminate teeth, and flagelliform branches (Figure 3). However, homoplasy cannot be discarded because several independent occurrences of these traits could also explain the observed pattern. C) Finally, three characters are symplesiomorphies, ancestral states that have been lost in several lineages during the evolutionary history of Orthotrichoideae: dioecy, development of connective membrane in the endostome, and presence of brood bodies (Figures 2, 3). The lack of autapomorphic morphological traits complicates genera delimitation within the subfamily. This, together with the overall homogeneity in the appearance of the plants of many different species, probably explains historical confusions in the taxonomy of the group. Similar results have been obtained in other groups of mosses, which reveals the flawed role of some morphological traits traditionally used for taxa delimitation. Such is the case of the sporophyte for the classification of the Funariaceae Schwägr. (Liu et al., 2012), the exostome ornamentation in Daltoniaceae Schimp. (Ho et al., 2012), or leaf characters in Polytrichales Cavers (Hyvönen et al., 2004) and Neckeraceae Schimp. (Olsson et al., 2009).

These results confirm our initial hypothesis that there is a general lack of autapomorphies that characterize supra-specific taxa in Orthotrichoideae. Nevertheless, the combination of a few of the analyzed features can still be used as diagnostic for certain groups. Creeping growth (plagiotropous) is characteristic in most of the Macromitrioideae, whereas all the species in Orthotrichoideae are acrocarpous mosses that commonly present main stems with erect growth. Creeping habit is then the exception in the subfamily, although it is characteristic in *Pentastichella* (Goffinet et al., 2004; Calabrese, 2006). In *Ulota*, this feature is also frequent and appears several times in the two main subclades, but is absent in the related genera *Plenogemma* and *Atlantichella* (Figure 2A).

Regarding sexual condition, both autoecy and dioecy are well represented in the family (Lewinsky, 1977; Goffinet and Vitt, 1998), albeit dioecy is quite rare among Orthotricheae, and characterizes the recently segregated genera *Nyholmiella*, *Plenogemma*, and *Pulvigera* (Goffinet et al., 2004; Plášek et al., 2015). Most genera within this tribe are either dioicous or autoicous, and only *Stoneobryum*, with only two species, includes both types of sexual condition. Conversely, dioecy is more frequent in Zygodonteae, although both *Zygodon* s. str. and *Codonoblepharon* comprise several monoicous species (see Malta, 1926).

As for stomata architecture, all Orthotrichaceae are phaneroporous, except two cryptoporous genera of Orthotricheae, *Orthotrichum* s. str. and *Stoneobryum*. Whether immersed stomata arose one or more times is a recurrent issue that has been suggested by several authors (Vitt, 1973; Lewinsky, 1977; Lewinsky-Haapasaari and Hedenäs, 1998[1999]; Goffinet et al., 2004), but its solution is still pending. Our reconstruction supports the result obtained by Goffinet et al. (2004), who suggested that immersed stomata would have evolved twice, one in *Orthotrichum* and the other in *Stoneobryum* (Figure 2C).

In Zygodonteae, crisped leaves are widely common in *Codonoblepharon*, although some of its species not included in the present study have leaves erect when dry. Conversely, these are only occasional within *Pentastichella* and *Zygodon*. In Orthotricheae, crisped leaves are common, although uneven on the subtribes: it is a rare trait in Orthotrichinae, albeit it is characteristic of *Stoneobryum* (Norris and Robinson, 1981) and *Sehnemobryum*, and occurs in at least two independent lineages of *Orthotrichum*. One of these lineages contains four of the five species included in this study with this character (Figure 2D). Among them, *O. pulchellum* Brunt. and *O. columbicum* Mitt. were outlined by Vitt (1971) as components of the subgenus *Pulchellum* (Schimp.) Vitt. Our phylogenetic reconstruction does not support the segregation of this lineage at the subgenus level, although a wider study could support its differentiation at section level. On the other hand, crisped leaves are frequent in Lewinskyinae and can be considered characteristic of *Atlantichella*, *Plenogemma*, and *Ulota*, although in the latter genus some exceptions occur in different lineages. Conversely, it is not frequent in *Lewinskya*, where crisped leaves occur in only three of the studied taxa.

The presence of dimorphic longitudinal bands of basal cells constitutes an exclusive character in two lineages of Zygodonteae: *Pentastichella* and *Australoria*, and in two genera of the subtribe Lewinskyinae in Orthotricheae: *Lewinskya* (Lara et al., 2016) and *Pulvigera* (Lara et al., 2020; Figure 2E). This character is especially apparent in *Pentastichella*, where the basal cells are strongly dimorphic, which is especially visible in cross section, and is more or less evident in the different species of the remaining genera where it has been observed. Other morphological characteristics of the basal cells have revealed to be important systematic features within Orthotrichoideae, as there are other characters related to them that can be used to define certain lineages. Basal marginal cells of leaves hyaline, short, with thickened walls characterize *Ulota* s. l. (Goffinet and Vitt, 1998). The phylogenetic reconstruction achieved in this study confirms that this is a defining character for three genera of Lewinskyinae (*Atlantichella*, *Plenogemma* and *Ulota*). Noteworthy, it also has been found in one *Orthotrichum* species, *O. calvum* (Figure 3E). The morphotype of this species resembles those of *Ulota* in several aspects, and presents a discordant number of chromosomes, in comparison to other species in this genus (Ramsay and Lewinsky, 1984). These deviating characteristics could be explained by evolutionary processes including intergeneric hybridization, but further studies are needed to verify this hypothesis. Lara et al. (2020) drew attention to the particular differentiation of denticulate-papillose marginal cells at the leaves base of the species of *Pulvigera*. This character is exclusive of *Atlantichella*, *Plenogemma*, and *Pulvigera* (Figure 3F). In both *Plenogemma* and *Pulvigera* it is evident, but it is not so clear in *Atlantichella*, where it can be better observed in the transition from the basis to the leaf blade. Finally, a band of elongate cells ascending from the transition base-blade some way up near leaf margins has long been considered a unique trait to *Ulota* (*Atlantichella*) *calvescens*, although it has also been recently described for *Plenogemma phyllantha* (Caparrós et al., 2014). Our molecular phylogenetic reconstruction confirms this character as exclusive of the two genera *Atlantichella* and *Plenogemma* (Figure 3D), and suggests that it would have been lost in *Ulota*, although Caparrós (2015) indicates that it could still be present in *Ulota robusta* Mitt. In *Atlantichella* it is an evident character that can be used to define the genus, whereas in *Plenogemma* it is usually less obvious.

Regarding gametophyte characters related to vegetative reproduction, brood bodies have been reported from several genera of the subfamily Orthotrichoideae, and their development has been considered characteristic of the small dioicous genera *Nyholmiella* and *Plenogemma* (Sawicki et al., 2017). Brood bodies also appear in one species of the dioicous genus *Pulvigera* (Lara et al., 2020), in most species (both dioicous or monoicous) of *Zygodon* and *Codonoblepharon*, and in many species of the autoicous *Orthotrichum* (Lewinsky, 1993). It has been stated that the presence of brood bodies is linked to dioicous lineages (Lewinsky, 1977; Sawicki et al., 2017), but our reconstruction does not confirm this (Figure 2F). Brood bodies are developed in the two tribes of the subfamily, and their absence from *Pentastichella*, *Stoneobryum*, *Sehnemobryum*, *Ulota* s. str., *Atlantichella*, and *Lewinskya* is significant. In *Orthotrichum* it is a very frequent character in some lineages, but it is infrequent in others, and it seems to be an ancestral character that has been lost in several species of the genus. Another form of vegetative reproduction documented in the family is clonal growth by means of stoloniferous-flagelliform branches. This has been shown to characterize *Pulvigera* (Lara et al., 2020), although it has also been reported from *Orthotrichum* and *Lewinskya* (Lara and Garilleti, 2014; Hugonnot, 2017). In the latter, it is a widespread character, although it has not been observed in some species (Figure 3G). It is also present in some *Ulota*, although in this genus the flagelliform branches are very short, and it seems that to a large degree, this genus has substituted this type of expansion for the reptant growth.

Within the sporophyte, the occurrence of the endostome connective membrane has been analyzed (Figure 3A). This structure drew little attention until Lewinsky (1993) considered it an important character to establish relationships within *Orthotrichum* s. l. Later, it has been highlighted as a characteristic peristomial element of *Orthotrichum* s. str. (Lara et al., 2016) and *Pulvigera* (Lara et al., 2020), but not in *Lewinskya*. The presence of a connective membrane has also been reported for species of other genera (e.g., Calabrese, 2006; Lara and Garilleti, 2014). It seems to be an ancestral character that is present in all the large lineages, and it is apparently only totally absent in *Stoneobryum* and *Sehnemobryum* (in Orthotrichinae), as well as in *Atlantichella*, *Plenogemma*, and *Lewinskya* (in Lewinskyinae), although in the latter genus samples with partial basal membrane have been reported (Lara et al., 2018). In *Ulota* it had not been observed until recently (Garilleti et al., 2020), and this study reveals its occurrence in several other species of the main lineages of the genus (Figure 3A and Supplementary Appendix 3).

Another important systematic character is the capsule striation (Figure 3B). Ribbed capsules are characteristic of most of Orthotrichaceae, and only some species in several genera show smooth capsules. Venturi (1887), Hagen (1908), Vitt (1971), and Lewinsky (1977, 1993) considered it a relevant character for *Orthotrichum* s. l. at the section level, and recently Lara et al. (2016) drew attention to the high frequency of smooth capsules in *Lewinskya*, where it is present in almost half of the species included in this analysis (Figure 3B). In contrast, only one of the studied species of *Orthotrichum* s. str. shows unribbed capsules.

Finally, the occurrence of multicellular spores has been traced (Figure 3C). Large, multicellular spores have been reported from several groups of Orthotrichaceae (Garilleti et al., 2012). This type of spores has been considered characteristic of *Matteria* (Macromitrioideae). In Orthotrichoideae they have been used to characterize *Muelleriella* (Vitt, 1976). Our analysis confirms the position of *Muelleriella* in *Orthotrichum* as proposed by Goffinet et al. (2004). The occurrence of multicellular spores is thus mostly limited to the two big genera *Orthotrichum* and *Ulota*, although it also has been reported in two *Lewinskya* species (Lewinsky-Haapasaari, 1995).

### Nomenclatural Changes

Family Orthotrichaceae Arn.

Subfamily Orthotrichoideae Broth.

Tribe Orthotricheae Engler

   Subtribe Orthotrichinae F.Lara, Garilleti & Draper nova

      Type: *Orthotrichum* Hedw.

      Genera: *Orthotrichum*, *Sehnemobryum*, *Stoneobryum*, and *Nyholmiella*

   Subtribe Lewinskyinae F.Lara, Garilleti & Draper nova

      Type: *Lewinskya* F.Lara, Garilleti & Goffinet

      Genera: *Lewinskya*, *Pulvigera*, *Plenogemma*, *Ulota*, and *Atlantichella*

      *Atlantichella* F.Lara, Garilleti & Draper gen. nov. (see sect. 4.4 for a description)

         Type: *Atlantichella calvescens* (Carrington) F.Lara, Garilleti & Draper

         *Atlantichella calvescens* (Carrington) F.Lara, Garilleti & Draper, comb. nov.

         ≡ *Ulota calvescens* Carrington, Bryoth. Eur. 11: 520. 1862

Tribe Zygodonteae Engler

      Genera: *Zygodon*, *Codonoblepharon*, *Pentastichella*, and *Australoria*

*Pentastichella* Müll.Hal. ex Thér.

         *Pentastichella chilensis* (Broth.) F.Lara, Garilleti & Draper, comb. nov.

         ≡ *Pleurorthotrichum chilense* Broth., Öfvers. Finska Vetensk.-Soc. Förh. 47(15): 1. 1–15. 1905

      *Australoria* F.Lara, Garilleti & Draper gen. nov. (see sect. 4.2 for a description)

      Type: *Australoria bartramioides* (Dusén ex Malta) F.Lara, Garilleti & Draper

         *Australoria bartramioides* (Dusén ex Malta) F.Lara, Garilleti & Draper, comb. nov.

         ≡ *Zygodon bartramioides* Dusén ex Malta, Latv. Univ. Raksti 10: 322. 1924

*Australoria chilensis* (Calabrese & F.Lara) F.Lara, Garilleti & Draper, comb. nov.

         ≡ *Zygodon chilensis* Calabrese & F.Lara, J. Bryol. 28: 97. 2006

## Data Availability Statement

The datasets presented in this study can be found in online repositories. The names of the repository/repositories and accession number(s) can be found in the article/Supplementary Material.

## Author Contributions

ID, FL, VM, and RG designed the research. ID, FL, RG, BV, and MF selected and processed the specimens for the molecular analyses. FL and RG selected and processed the specimens for the morphological study. ID, FL, BV, JC, and MF contributed to the phylogenetical analyses. ID, JC, and BV performed the dating analyses. ID, FL, and RG performed the analyses of the morphological characters evolution. ID, FL, and RG prepared the illustrations. ID and FL wrote a first draft of the manuscript. All the authors contributed in writing the final version of the manuscript.

## Conflict of Interest

The authors declare that the research was conducted in the absence of any commercial or financial relationships that could be construed as a potential conflict of interest.

## Footnotes

1 www.macrogen.com

2 https://www.geneious.com
