# Supplementary material for: Insights Into the Evolutionary History of the Subfamily Orthotrichoideae (Orthotrichaceae, Bryophyta): New and Former Supra-Specific Taxa So Far Obscured by Prevailing Homoplasy
Source: Front Plant Sci. 2021 Mar 26;12:629035. doi: 10.3389/fpls.2021.629035 (PMC8034389; doi:10.3389/fpls.2021.629035)
Supplement: Supplementary file 1 [file Data_Sheet_1.docx]

Appendix 1. Details of samples included in the molecular analyses: Taxon; Country; Herbarium voucher; DNA code; GenBank (<http://www.ncbi.nlm.nih.gov>) accession numbers (*ITS*2*, rps*4*, trn*G*, trn*L-F). The * beside the DNA code indicates those samples whose DNA was extracted for this study. The rest of the DNA was extracted following the protocols by: ^1^ Goffinet et al. (2004), ^2^ Vigalondo et al. (2016), ^3^ Caparrós et al. (2016), ^4^ Lara et al. (2020), ^5^ Medina et al. (2013), ^6^ Patiño et al. (2013). GenBank accession numbers in italics correspond to sequences newly obtained for this study.

*Codonoblepharon forsteri* (Dicks.) Goffinet; Spain, Álava; MAUAM-1950; ID384*; (*MW264075*, *MW401280*, *MW401362*, *MW401442*). *Codonoblepharon forsteri* (Dicks.) Goffinet; Spain, Málaga; MAUAM-4956; ID397^4^; (MN529593, MN596378, MN542450, MN542426). *Codonoblepharon menziesii* Schwägr*.*; Unknown; BG Shevock 18773; 1603^1^; (--, *MW401281*, *MW401363*, *MW401443*). *Leratia obtusifolia* (Hook.) Goffinet; Unknown; Allen 12284; 798^1^; (*MW264076*, *MW401282*, *MW401364*, *MW401444*). *Lewinskya acuminata* (H. Philib.) F. Lara, Garilleti & Goffinet; Spain, Ávila; MAUAM-Brio 3272; BV010^2^; (KT862263, KT862293, MN542451, KT862322). *Lewinskya affinis* (Brid.) F. Lara, Garilleti & Goffinet; Spain, Burgos; MAUAM-Brio 4329; O108^5^; (MN529594, JQ836796, JQ836900, JQ836985). *Lewinskya arborescens* (Thér. & Naveau) F. Lara, Garilleti & Goffinet; Tanzania, Kilimanjaro; MAUAM-Brio 4590; BV070^4^; (MN529595, MN177279, MN542452, MN542427). *Lewinskya bolanderi* (Sull.) F. Lara, Garilleti & Goffinet; USA, California; MAUAM-Brio 4572; BV059^4^; (*MW264077*, MN177281, *MW401365*, *MW401445*). *Lewinskya breviseta* (F. Lara, Garilleti & Mazimpaka) F. Lara, Garilleti & Goffinet; Turkey; MAUAM-Brio 4450; BV017^2^; (KT862285, KT862314, *MW401366*, KT862343). *Lewinskya cyathiformis* (R. Br. bis) F. Lara, Garilleti & Goffinet; Unknown; BG Schäfer-Verwimp 14157; 1187^1^; (*MW264078*, *MW401283*, *MW401367*, *MW401446*). *Lewinskya dasymitria* (Lewinsky) F. Lara, Garilleti & Goffinet; China, Yunnan; E-Long 18562; 1167^1^; (*MW264079*, *MW401284*, *MW401368*, *MW401447*). *Lewinskya elongata* (Taylor) F. Lara, Garilleti & Goffinet; Unknown; Steere & Balslev 26093; 1034^1^; (MN529596, MN596379, MN542453, MN542428). *Lewinskya erosa* (Lewinsky) F. Lara, Garilleti & Goffinet; Unknown; MO Allen 7183; 1609^1^; (*MW264080*, *MW401285*, *MW401369*, *MW401448*). *Lewinskya firma* (Venturi) F. Lara, Garilleti & Goffinet; Unknown; MO Petelin 28-18; 1611^1^; (*MW264081*, *MW401286*, *MW401370*, *MW401449*). *Lewinskya holzingeri* (Renauld & Cardot) F. Lara, Garilleti & Goffinet; USA, Wyoming; DUKE-Vitt Exs. 50; 791^1^; (*MW264082*, *MW401287*, *MW401371*, *MW401450*). *Lewinskya hookeri* (Wilson ex Mitt.) F. Lara, Garilleti & Goffinet; Unknown; E-Long 24184; 1169^1^; (*MW264083*, *MW401288*, *MW401372*, *MW401451*). *Lewinskya hortensis* (Bosw.) F. Lara, Garilleti & Goffinet; Unknown; BG Schäfer-Verwimp 141156; 1188^1^; (*MW264084*, *MW401289*, *MW401373*, *MW401452*). *Lewinskya iberica* (F. Lara & Mazimpaka) F. Lara, Garilleti & Goffinet; Spain, Ávila; MAUAM-Brio 4567; BV056^4^; (MN529597, MN177247, MN542454, MN542429). *Lewinskya incana* (Müll. Hal.) F. Lara, Garilleti & Goffinet; Chile, Aysen del General Carlos Ibañez del Campo; MAUAM-Brio 4576; BV079^4^; (MN529598, MN177272, MN542455, MN542430). *Lewinskya laevigata* (J.E. Zetterst.) F. Lara, Garilleti & Goffinet; USA, Nevada; MAUAM-Brio; JA055-1*; (*MW264085*, *MW401290*, *MW401374*, *MW401453*). *Lewinskya ludificans* (Lewinsky) F. Lara, Garilleti & Goffinet; Unknown; CONN Goffinet 5488; 1028^1^; (*MW264086*, *MW401291*, *MW401375*, *MW401454*). *Lewinskya mandonii* (Schimp. ex Hampe) F. Lara, Garilleti & Goffinet; Bolivia, La Paz; MAUAM-Brio 4573; BV081^4^; (*MW264087*, MN177248, *MW401376*, *MW401455*). *Lewinskya praemorsa* (Venturi) F. Lara, Garilleti & Goffinet; USA, Nevada; MAUAM-Brio 4570; BV060^4^; (*MW264088*, MN177265, *MW401377*, *MW401456*). *Lewinskya pycnophylla* (Schimp.) F. Lara, Garilleti & Goffinet; USA, New Mexico; DUKE-Worthington 31070; 1503^1^; (*MW264089*, *MW401292*, --, *MW401457*). *Lewinskya pylaisii* (Brid.) F. Lara, Garilleti & Goffinet; Unknown; Vandenpoorten 27/07/99; 1110^1^; (*MW264090*, *MW401293*, --, *MW401458*). *Lewinskya rupestris* (Schleich. ex Schwägr.) F. Lara, Garilleti & Goffinet; Spain, Ávila; VAL-Briof 7043; ID367*; (*MW264091*, *MW401294*, *MW401378*, *MW401459*). *Lewinskya sainsburyi* (Allison) F. Lara, Garilleti & Goffinet; New Zealand, South Island; MAUAM-Brio 2103; BV084^4^; (*MW264092*, MN177276, --, *MW401460*). *Lewinskya shawii* (Wilson) F. Lara, Garilleti & Goffinet; Greece, Ipiros; MAUAM-Brio 4587; BV068^4^; (*MW264093*, MN177270, *MW401379*, *MW401461*). *Lewinskya sordida* (Sull. & Lesq.) F. Lara, Garilleti & Goffinet; Unknown; Belland 17792; 1057^1^; (*MW264094*, *MW401295*, *MW401380*, *MW401462*). *Lewinskya spanotricha* (Lewinsky) F. Lara, Garilleti & Goffinet; Unknown; NY isotype Schäfer-Verwimp 12214; 1222^1^; (*MW264095*, --, --, *MW401463*). *Lewinskya speciosa* (Nees) F. Lara, Garilleti & Goffinet; USA, California; MAUAM-Brio 4452; BV019^2^; (KT862281, KT862312, MN542456, KT862341). *Lewinskya striata* (Hedw.) F. Lara, Garilleti & Goffinet; Turkey, Antalya; MAUAM-Brio 4446; BV013^2^; (KT862287, KT862316, *MW401381*, KT862345). *Lewinskya tanganyikae* (P. de la Varde) F. Lara, Garilleti & Goffinet; Kenya, Mt. Kenya; MAUAM-Brio 4593; BV075^4^; (*MW264096*, MN177257, *MW401382*, *MW401464*). *Lewinskya tasmanica* (Hook. f. & Wilson) F. Lara, Garilleti & Goffinet; New Zealand, North Island; MAUAM-Brio; ID394*; (*MW264097*, *MW401296*, *MW401383*, *MW401465*). *Lewinskya tasmanica* var. *parvitheca* (R. Br. bis) F. Lara, Garilleti & Goffinet; Unknown; BG Schäfer-Verwimp 13833; 1189^1^; (*MW264098*, *MW401297*, *MW401384*, *MW401466*). *Lewinskya tortidontia* (F. Lara, Garilleti & Mazimpaka) F. Lara, Garilleti & Goffinet; Turkey, Antalya; MAUAM-Brio 4603; BV098^4^; (*MW264099*, MN177267, *MW401385*, *MW401467*). *Lewinskya vladikavkana* (Venturi) F. Lara, Garilleti & Goffinet; Russia, Altai; MHA-Ignatov 34/10; 1184^1^; (*MW264100*, *MW401298*, *MW401386*, *MW401468*). *Macrocoma lycopodioides* (Schwägr.) Vitt; South Africa, Western Cape; MAUAM-Brio 2953; BV024^2^; (KT862258, KT862288, --, KT804333). *Nyholmiella gymnostoma* (Bruch ex Brid.) Holmen & E. Warncke; Canada, Newfoundland and Labrador; NY Zander 4809; 1219^1^; (--, *MW401299*, *MW401387*, *MW401469*). *Nyholmiella obtusifolia* (Brid.) Holmen & E. Warncke; Spain, Burgos; MAUAM-Brio 4343; O118^5^; (MN529599, JQ836797, JQ836901, JQ836986). *Orthotrichum alpestre* Hornsch. ex Bruch & Schimp.; Unknown; MAUAM-Brio; R669^5^; (MH275451, JQ836864, JQ836967, JQ837053). *Orthotrichum anomalum* Hedw.; Spain, Asturias; MAUAM-Brio 4330; O120^5^; (MH275453, JQ836799, JQ836903, JQ836988). *Orthotrichum assimile* Müll. Hal.; Unknown; CONN Goffinet 5621; 1429^1^; (*MT902366*, AY618364, *MW401388*, AY636020). *Orthotrichum bartramii* R. S. Williams; USA, Arizona; CAS Kellman 5457; Shaw, Shevock & Spence; R598^5^; (MH275454, JQ836838, JQ836942, JQ837027). *Orthotrichum callistomum* Fisch.-Oost. ex Bruch & Schimp.; Nepal, Sagarmatha; MAUAM-Brio 3403; FL002*; (*MT902367*, *MW401300*, *MW401389*, *MW401470*). *Orthotrichum callistomum* Fisch.-Oost. ex Bruch & Schimp.; Nepal, Bagmati; MAUAM-Brio 3404; FL004*; (*MW264101*, *MW401301*, *MW401390*, *MW401471*). *Orthotrichum calvum* Hook. f. & Wilson; Unknown; MO Fife & Espie 8230; 1614^1^; (*MT902368*, *MW401302*, *MW401391*, *MW401472*). *Orthotrichum casasianum* F. Lara, Garilleti & Mazimpaka; Spain, Álava; MAUAM-Brio 1702; R398^5^; (MN529600, JQ836811, JQ836915, JQ837000). *Orthotrichum columbicum* Mitt*.*; Spain, León; MAUAM-Brio 657; R678^5^; (MH275458, JQ836877, JQ836977, JQ837066). *Orthotrichum comosum* F. Lara, R. Medina & Garilleti; Spain, Cádiz; MAUAM-Brio 4361; R673^5^; (MH275460, JQ836860, JQ836964, JQ837049). *Orthotrichum confusum* R. Medina, F. Lara & Garilleti; USA, California; MAUAM-Brio 4323; R680^5^; (MH275463, JQ836878, JQ836978, JQ837067). *Orthotrichum consimile* Mitt.; USA, California; UC-1760062; R616^5^; (MN529601, JQ836870, JQ836971, JQ837059). *Orthotrichum coulteri* Mitt*.*; USA, California; MAUAM-Brio 4366; R564^5^; (MH275464, JQ836817, JQ836921, JQ837006). *Orthotrichum crassifolium* Hook.f. & Wilson; Chile, Cabo de Hornos; MAUAM-Brio 4963; ID402*; (*MW264102*, *MW401317*, --, --). *Orthotrichum crassifolium* Hook.f. & Wilson; New Zealand, South Island; MAUAM-Brio 4964; ID403*; (*MT902381*, *MW401318*, *MW401403*, --). *Orthotrichum crassifolium* Hook.f. & Wilson; Chile, Cabo de Hornos; MAUAM-Brio 3184; ID417*; (*MW264103*, *MW401319*, --, --). *Orthotrichum crenulatum* Mitt.; Kazakhstan; MO Magill 10295; 1617^1^; (*MT902382*, *MW401320*, *MW401404*, *MW401486*). *Orthotrichum crispifolium* Broth.; China; E-Long 24516; 1168^1^; (*MT902383*, *MW401321*, *MW401405*, *MW401487*). *Orthotrichum cucullatum* F. Lara, R. Medina & Garilleti; USA, California; UC-1768584; R579^5^; (MH275467, JQ836832, JQ836936, JQ837021). *Orthotrichum cupulatum* Brid.; Unknown; Sauer 08/01/92; 1105^1^; (--, JQ836886, --, JQ837075). *Orthotrichum cupulatum* var. *riparium* Huebener; Unknown; Sauer 25.06.90; 1108^1^; (*MT902384*, JQ836893, *MW401406*, JQ837082). *Orthotrichum diaphanum* Brid.; Spain, Madrid; MAUAM-Brio 4565; 3917^1^; (*MT902385*, *MW401322*, *MW401407*, *MW401488*). *Orthotrichum franciscanum* F. Lara, R. Medina & Garilleti; USA, California; MAUAM-Brio 4390; R570^5^; (MH275468, JQ836823, JQ836927, JQ837012). *Orthotrichum gigantosporum* Lewinsky; Chile, Aysén; RG2016-236g; RG023*; (*MW264104*, *MW401323*, *MW401408*, *MW401489*). *Orthotrichum handiense* F. Lara, Garilleti & Mazimpaka; Spain, Canary Islands; MAUAM-Brio; O2115^6^; (MH275472, JX297214, JX297219, *MW401490*). *Orthotrichum macrocephalum* F. Lara, Garilleti & Mazimpaka; Spain, Jaen; MAUAM-Brio 4463; BV104^4^; (MN529602, MN596380, MN542457, MN542431). *Orthotrichum norrisii* F. Lara, R. Medina & Garilleti; USA, California; MAUAM-Brio 4395; R567^5^; (MN529603, JQ836820, JQ836924, JQ837009). *Orthotrichum pellucidum* Lindb.; Unknown; CONN Goffinet 8217; 1277^1^; (*MT902386*, *MW401324*, *MW401409*, --). *Orthotrichum persimile* F. Lara, R. Medina & Garilleti; USA, California; UC-1650645; R580^5^; (MH275479, JQ836833, JQ836937, JQ837022). *Orthotrichum pilosissimum* R. Medina, F. Lara & Garilleti; USA, Nevada; MAUAM-Brio 4334; R640^5^; (MH275481, JQ836845, JQ836949, JQ837034). *Orthotrichum pulchellum* Brunt.; Canada, British Columbia; MAUAM-Brio 4336; R682^5^; (MH275483, JQ836880, JQ836980, JQ837069). *Orthotrichum pusillum* Mitt.; USA, Connecticut; DUKE-Goffinet 8152; 1230^1^; (--, *MW401325*, *MW401410*, *MW401491*). *Orthotrichum scanicum* Grönvall; Greece, Sterea Hellada; MAUAM-Brio 2166; R018^5^; (MH275485, JQ836800, JQ836904, JQ836989). *Orthotrichum schimperi* Hammar; Tunisia, Aïn-Draham; MAUAM-Brio 2448; R364^5^; (MN529604, JQ836810, JQ836914, JQ836999). *Orthotrichum sharpii* H. Rob.; Mexico, Veracruz; MAUAM-Brio 4340; R679^5^; (*MT902387*, JQ836861, JQ836965, JQ837050). *Orthotrichum shevockii* Lewinsky & D.H. Norris; USA, California; MAUAM-Brio 3280; BV043^4^; (MH275491, MH275513, *MW401411*, MH275533). *Orthotrichum stellatum* Brid.; USA, New England; CONN Goffinet 10579; 2739^1^; (*MT902388*, *MW401326*, *MW401412*, *MW401492*). *Orthotrichum strangulatum* P. Beauv.; Unknown; Redfearn Exs. 207; 818^1^; (*MT902389*, *MW401327*, *MW401413*, *MW401493*). *Orthotrichum subexsertum* Schimp. ex Müll. Hal.; Unknown; Magill 4026; 1050^1^; (--, *MW401328*, *MW401414*, *MW401494*). *Orthotrichum tenellum* Bruch ex Brid.; Italy, Sicily; MAUAM-Brio 4346; R294^5^; (MN529605, JQ836805, JQ836909, JQ836994). *Orthotrichum underwoodii* F. Lara, Garilleti & Mazimpaka ; USA, California; MAUAM-Brio 4341; R583^5^; (MH275503, JQ836835, JQ836939, JQ837024). *Orthotrichum vittii* F. Lara, Garilleti & Mazimpaka; Spain, cf. Soria; Lara & Vergara; 1115^1^; (*MT902390*, *MW401329*, *MW401415*, *MW401495*). *Pentastichella pentasticha* (Mont.) Müll. Hal. ex Thér.; Argentina, Córdoba; MAUAM-Brio 2981; ID207/BV038^2^; (KT862259, KT862289, *MW401416*, KT862318). *Plenogemma phyllantha* (Brid.) Sawicki, Plášek & Ochyra; USA, Washington; MAUAM-Brio; ID331^3^; (KT804291, *MW401330*, KT804330, KT804370). *Plenogemma phyllantha* (Brid.) Sawicki, Plášek & Ochyra; Canada, British Columbia; MAUAM-Brio; ID386*; (*MW264105*, *MW401331*, *MW401417*, *MW401496*). *Plenogemma phyllantha* (Brid.) Sawicki, Plášek & Ochyra; United Kingdom, England; MAUAM-Brio 2911; ID387*; (*MW264106*, *MW401332*, *MW401418*, *MW401497*). *Plenogemma phyllantha* (Brid.) Sawicki, Plášek & Ochyra; USA, Washington; MAUAM-Brio; ID388*; (*MW264107*, *MW401333*, *MW401419*, *MW401498*). *Pleurorthotrichum chilense* Broth.; Unknown; CONN Goffinet 5320; 959^1^; (*MW264108*, *MW401334*, *MW401420*, *MW401499*). *Pulvigera howei* (Renauld & Cardot ) F.Lara, Draper & Garilleti; USA, California; MAUAM-Brio 4453; BV020^2^; (KT862283, KT862311, MN542467, KT862340). *Pulvigera lyellii* (Hook. & Taylor) Plášek, Sawicki & Ochyra; USA, California; MAUAM-Brio 4451; BV018^2^; (KT862282, KT862310, MN542458, KT862339). *Pulvigera papillosa* (Hampe) F. Lara, Draper & Garilleti; Canada, British Columbia; MAUAM-Brio; ID409; (MN529620, MN596395, MN542474, MN542446). *Pulvigera pringlei* (Müll. Hal.) F. Lara, Draper & Garilleti; USA, California; MAUAM-Brio; ID407; (MN529616, MN596391, MN542470, MN542442). *Sehnemobryum paraguense* (Besch.) Lewinsky & Hedenäs; Unknown; ALTA Vitt 21087; 1516^1^; (*MW264109*, AY618382, *MW401421*, AY636013). *Stoneobryum mirum* (Lewinsky) D.H. Norris & H. Rob.; South Africa, Eastern Cape; DUKE-Vanderpoorten 156; 1506^1^; (*MW264110*, AY618381, *MW401422*, AY636012). *Ulota billbuckii* Garilleti, Mazimpaka & F. Lara; Chile, Aysén; RG2017-012d; RG020; (--, *MW401335*, *MW401423*, *MW401500*). *Ulota bruchii* Hornsch. ex Brid.*.*; Spain, Asturias; MAUAM-Brio 4444; ID383^3^; (KT804259, *MW401336*, KT804298, KT804337). *Ulota calvescens* Carrington; Spain, Canary Islands; MAUAM-Brio; ID199*; (*MW264111*, *MW401337*, *MW401424*, *MW401501*). *Ulota calvescens* Carrington; Spain, Cádiz; MAUAM-Brio; ID393*; (*MW264112*, *MW401338*, *MW401425*, *MW401502*). *Ulota calvescens* Carrington; Spain, Canary Islands; MAUAM-Brio 4957; ID399*; (*MW264113*, *MW401339*, *MW401426*, *MW401503*). *Ulota calvescens* Carrington; Spain, Asturias; MAUAM-Brio 4442; ID400*; (*MW264114*, *MW401340*, *MW401427*, *MW401504*). *Ulota calvescens* Carrington; Spain, Cádiz; MAUAM-Brio 4443; ID401*; (*MW264115*, *MW401341*, *MW401428*, *MW401505*). *Ulota carinata* Mitt*.*; Chile, Aysén; RG2016-237f; RG027*; (*MW264116*, *MW401342*, *MW401429*, *MW401506*). *Ulota coarctata* (P. Beauv.) Hammar; Spain, Cantabria; MAUAM-Brio 4438; ID360^3^; (KT804260, MN596397, KT804299, KT804338). *Ulota crispa* (Hedw.) Brid.; Romania, Pietroase; MAUAM-Brio; ID320^3^; (KT804270, *MW401343*, KT804309, KT804348). *Ulota crispula* Bruch; Ireland, Wicklow; MAUAM-Brio 4814; ID355^3^; (KT804275, MN596398, KT804314, KT804353). *Ulota curvifolia* (Wahlenb.) Sw.; Russia, Mt. Ural; MAUAM-Brio 4826; ID359^3^; (KT804278, MN596399, KT804317, KT804357). *Ulota drummondii* (Hook. & Grev.) Brid.; Japan, Hokkaido; MAUAM-Brio; ID356^3^; (KT804280, *MW401344*, KT804319, KT804359). *Ulota hutchinsiae* (Sm.) Hammar; USA, New England; CONN Goffinet 10580; 2740^1^; (*MW264117*, *MW401345*, *MW401430*, *MW401507*). *Ulota intermedia* Schimp.; Japan, Hokkaido; MAUAM-Brio 4817; ID343^3^; (KT804281, MN596400, KT804320, KT804360). *Ulota japonica* (Sull. & Lesq.) Mitt.; Canada, British Columbia; DUKE-Schofield 86158; 1136^1^; (*MW264118*, *MW401346*, *MW401431*, *MW401508*). *Ulota longifolia* Dixon & Sakurai; USA, Oregon; MAUAM-Brio; ID350*; (*MW264119*, *MW401347*, *MW401432*, *MW401509*). *Ulota macrodontia* Dixon & Malta; Chile, Aysén; RG2016-236a; RG025*; (*MW264120*, *MW401348*, *MW401433*, *MW401510*). *Ulota macrospora* Baur & Warnst.; France, Haute-Savoie; MAUAM-Brio; ID365^3^; (KT804288, *MW401349*, KT804327, KT804367). *Ulota magellanica* (Mont.) A. Jaeger; Unknown; CONN Goffinet 5886; 1045^1^; (MN529622, MN596401, MN542476, MN542448). *Ulota maltiana* Garilleti & F. Lara; Chile, Los Lagos; VAL-Briof 11812; RG010*; (*MW264121*, *MW401350*, *MW401434*, *MW401511*). *Ulota megalospora* Venturi; Canada, British Columbia; MAUAM-Brio; ID358^3^; (KT804289, *MW401351*, KT804328, KT804368). *Ulota obtusiuscula* Müll. Hal. & Kindb.; Canada, British Columbia; MAUAM-Brio; ID329^3^; (KT804290, *MW401352*, KT804329, KT804369). *Ulota perichaetialis* (Sainsbury) Goffinet; New Zealand, South Island; MAUAM-Brio 4955; ID395*; (*MW264122*, *MW401353*, *MW401435*, *MW401512*). *Ulota perichaetialis* (Sainsbury) Goffinet; New Zealand, North Island; MAUAM-Brio 4877; ID396*; (*MW264123*, *MW401354*, *MW401436*, *MW401513*). *Ulota pycnophylla* Dusén ex Malta; Unknown; CONN Goffinet 5547; 1031^1^; (MN529623, MN596402, MN542477, MN542449). *Ulota rehmannii* Jur*.*; Turkey, Trabzon; MAUAM-Brio; ID328^3^; (KT804292, *MW401355*, KT804331, KT804371). *Ulota reptans* Mitt*.*; Japan, Honshu; MAUAM-Brio; ID349^3^; (KT804293, *MW401356*, KT804332, KT804372). *Ulota streptodon* Garilleti, Mazimpaka & F. Lara; Chile, Aysen; RG2017-012e; RG022*; (*MW264124*, *MW401357*, *MW401437*, *MW401514*). *Zygodon bartramioides* Malta; Chile, Araucanía; DUKE-Goffinet 5476; 1009^1^; (--, AY618371, --, AY636007). *Zygodon campylophyllus* Müll. Hal.; Unknown; DUKE-Steere 23100; 1128^1^; (*MW264125*, *MW401358*, --, *MW401515*). *Zygodon hookeri* var. *leptobolax* (Müll. Hal.) Calabrese; Unknown; CONN Goffinet 5408 under *Zygodon inermis* Malta; 998^1^; (*MW264126*, *MW401359*, *MW401438*, *MW401516*). *Zygodon peruvianus* Sull*.*; Unknown; DUKE-Griffin PV-132 under *Zygodon goudotii* Hampe; 1131^1^; (*MW264127*, *MW401360*, *MW401439*, *MW401517*). *Zygodon rupestris* Schimp. ex Lorentz; China; Long 32510; Long 32510; (--, MH175803, --, MH175918). *Zygodon sibiricus* Ignatov, Ignatova, Z. Iwats. & B.C. Tan; Russia, Altai; MHA-Ignatov 1/82; 1183^1^; (*MW264128*, *MW401361*, *MW401440*, *MW401518*). *Zygodon viridissimus* (Dicks.) Brid.; United Kingdom, England; MAUAM-Brio 2910; ID208/BV037^2^; (KT862260, KT862290, *MW401441*, KT862319).
