# Supplementary material for: Insights Into the Evolutionary History of the Subfamily Orthotrichoideae (Orthotrichaceae, Bryophyta): New and Former Supra-Specific Taxa So Far Obscured by Prevailing Homoplasy
Source: Front Plant Sci. 2021 Mar 26;12:629035. doi: 10.3389/fpls.2021.629035 (PMC8034389; doi:10.3389/fpls.2021.629035)
Supplement: Supplementary file 2 [file Data_Sheet_2.docx]

Appendix 2. Alternative models used to estimate divergence times. The model highlighted in bold is the one selected as the best model, according to Bayes factors.

| **Model number** | **Model type** | **Speciation Tree-Prior** | **Substitution rate** | **PS mean** | **SS mean** |
| --- | --- | --- | --- | --- | --- |
| **1** | **Relaxed uncorrelated log-normal clock** | **Yule** | **Absolute = 5 x 10-4, stdev range 1.5 × 10–4** | **-226,16634** | **-226,16578** |
| 2 | Relaxed uncorrelated log-normal clock | Birth-Death | Absolute = 5 x 10-4, stdev range 1.5 × 10–4 | -293,4052343 | -293,4049821 |
| 3 | Relaxed uncorrelated log-normal clock | Coalescent: logistic growth | Absolute = 5 x 10-4, stdev range 1.5 × 10–4 | -1527,915039 | -1527,909711 |
| 4 | Relaxed uncorrelated log-normal clock | Yule | Plastid = 5.0E-4, stdev 1.5E-4 / Nuc = 1.35 × 10−3, stdev 1.0E-4 | -549,3645373 | -549,363595 |
| 5 | Relaxed uncorrelated log-normal clock | Birth-Death | Plastid = 5.0E-4, stdev 1.5E-4 / Nuc = 1.35 × 10−3, stdev 1.0E-4 | -549,1641684 | -549,1639653 |
| 6 | Relaxed uncorrelated log-normal clock | Coalescent: logistic growth | Plastid = 5.0E-4, stdev 1.5E-4 / Nuc = 1.35 × 10−3, stdev 1.0E-4 | -2484,653756 | -2484,655227 |
| 7 | Relaxed uncorrelated exponential | Yule | Absolute = 5 x 10-4, stdev range 1.5 × 10–4 | -471,5547038 | -471,5537244 |
| 8 | Relaxed uncorrelated exponential | Birth-Death | Absolute = 5 x 10-4, stdev range 1.5 × 10–4 | -404,9614027 | -404,9615673 |
| 9 | Relaxed uncorrelated exponential | Coalescent: logistic growth | Absolute = 5 x 10-4, stdev range 1.5 × 10–4 | -2437,6526 | -2437,64267 |
| 10 | Relaxed uncorrelated exponential | Yule | Plastid = 5.0E-4, stdev 1.5E-4 / Nuc = 1.35 × 10−3, stdev 1.0E-4 | -441,5232129 | -441,5235151 |
| 11 | Relaxed uncorrelated exponential | Birth-Death | Plastid = 5.0E-4, stdev 1.5E-4 / Nuc = 1.35 × 10−3, stdev 1.0E-4 | -419,6535783 | -419,6538383 |
| 12 | Relaxed uncorrelated exponential | Coalescent: logistic growth | Plastid = 5.0E-4, stdev 1.5E-4 / Nuc = 1.35 × 10−3, stdev 1.0E-4 | -1806,732136 | -1806,729775 |
| 13 | Relaxed uncorrelated log-normal clock | Yule | Continuous Time Markov Chain (CTMC) rate reference | -14117,38441 | -14116,78433 |
| 14 | Relaxed uncorrelated log-normal clock | Yule | ucld mean LogTransformedNormal | -14133,25414 | -14133,35152 |
| 15 | Relaxed uncorrelated log-normal clock | Yule | ucld mean Normal | -14122,02086 | -14121,74803 |
| 16 | Relaxed uncorrelated log-normal clock | Birth-Death | Continuous Time Markov Chain (CTMC) rate reference | -14114,89478 | -14114,23845 |
| 17 | Relaxed uncorrelated log-normal clock | Birth-Death | ucld mean LogTransformedNormal | -14130,46719 | -14130,90513 |
| 18 | Relaxed uncorrelated log-normal clock | Birth-Death | ucld mean Normal | -14117,50057 | -14117,33411 |
| 19 | Relaxed uncorrelated log-normal clock | Coalescent: logistic growth | Continuous Time Markov Chain (CTMC) rate reference | -14095,82432 | -14095,8542 |
| 20 | Relaxed uncorrelated log-normal clock | Coalescent: logistic growth | ucld mean Normal | -14093,74928 | -14093,86652 |
| 21 | Relaxed uncorrelated exponential | Yule | Continuous Time Markov Chain (CTMC) rate reference | -14121,52633 | -14121,28934 |
| 22 | Relaxed uncorrelated exponential | Yule | ucld mean Normal | -14125,8821 | -14125,28222 |
| 23 | Relaxed uncorrelated exponential | Birth-Death | Continuous Time Markov Chain (CTMC) rate reference | -14120,95764 | -14120,6717 |
| 24 | Relaxed uncorrelated exponential | Birth-Death | ucld mean LogTransformedNormal | -14144,38111 | -14144,59712 |
| 25 | Relaxed uncorrelated exponential | Birth-Death | ucld mean Normal | -14126,4198 | -14125,79256 |
| 26 | Relaxed uncorrelated exponential | Coalescent: logistic growth | Continuous Time Markov Chain (CTMC) rate reference | -14110,39153 | -14110,25988 |
| 27 | Relaxed uncorrelated exponential | Coalescent: logistic growth | ucld mean Normal | -14101,40107 | -14101,09982 |
