# Supplementary material for: Insights Into the Evolutionary History of the Subfamily Orthotrichoideae (Orthotrichaceae, Bryophyta): New and Former Supra-Specific Taxa So Far Obscured by Prevailing Homoplasy
Source: Front Plant Sci. 2021 Mar 26;12:629035. doi: 10.3389/fpls.2021.629035 (PMC8034389; doi:10.3389/fpls.2021.629035)

Appendix 4. Chronogram obtained from the dating analysis under a relaxed uncorrelated log-normal clock with Yule speciation and an absolute nucleotide substitution rate of $5x10^{-4}$, with stdev rangeof $1.5x10^{-4}$. Bayesian Posterior probabilities are shown above the branches, mean ages are shown at the right side of the nodes, and 95% highest posterior density intervals are indicated with coloured bars.

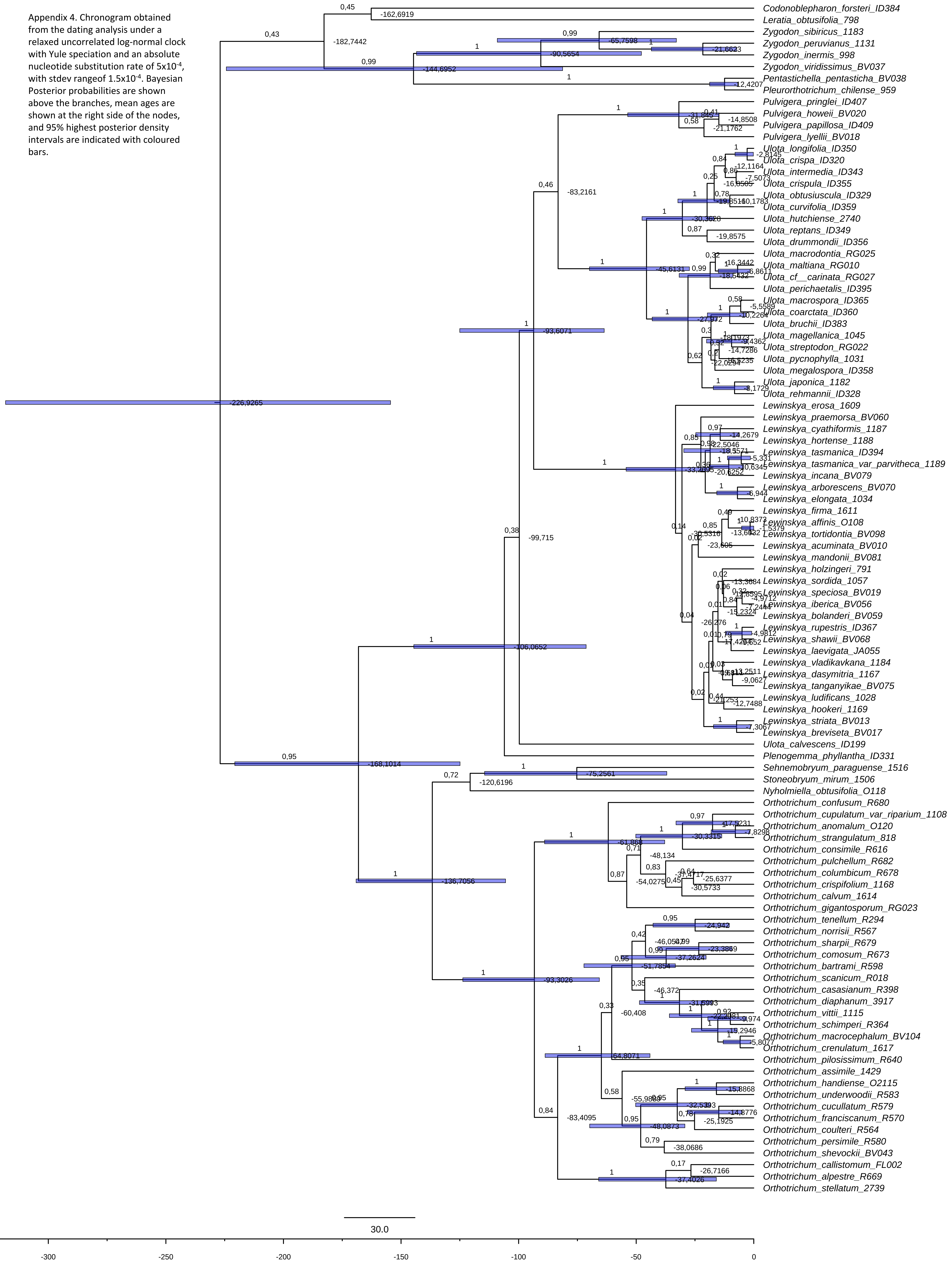

Supplement: Supplementary file 4 [file Data_Sheet_4.pdf]
